# Associations Between Experiences of Racial Discrimination Across the Life Course and Mental Health: Exploring Direct and Indirect Pathways

**DOI:** 10.1111/1467-9566.70023

**Published:** 2025-04-09

**Authors:** Patricia Irizar, Dharmi Kapadia, Harry Taylor, Gertrude Wafula, Charles Kwaku‐Odoi, Laia Bécares, Srinivasa Vittal Katikireddi

**Affiliations:** ^1^ Department of Sociology School of Social Sciences University of Manchester Manchester UK; ^2^ Department of Global Health & Medicine King's College London London UK; ^3^ Caribbean and African Health Network (CAHN) Manchester UK; ^4^ School of Nursing and Public Health Manchester Metropolitan University Manchester UK; ^5^ MRC/CSO Social & Public Health Sciences Unit University of Glasgow Glasgow UK

**Keywords:** common mental disorders, ethnic inequalities, ethnicity, health inequalities, mental health, racism

## Abstract

We aim to explore the association between racial discrimination across the life course on common mental disorders (CMD) during the COVID‐19 pandemic, testing direct and indirect pathways. Cross‐sectional data were obtained from the Evidence for Equality National Survey (Feb–Nov 2021, *N* = 8897 ethnic minority people aged 18–60). The survey measured experiences of racial discrimination across multiple domains and time periods. Path analyses were used to explore the associations between racial discrimination and CMD and the indirect associations via SARS‐CoV‐2 infection, financial concerns, loneliness and belonging. We find a clear dose–response relationship between experiences of racial discrimination over time and CMD. Compared to no reporting of experiences, chronic experiences of racial discrimination were associated with 2.91 times the odds of CMD (95%CI: 2.33–3.65; recent experiences only OR = 2.11, 1.67–2.67; past experiences only OR = 1.50, 1.16–1.92). Recent and chronic experiences of racial discrimination (but not past experiences) were also indirectly associated with CMD, via SARS‐CoV‐2 infection, greater financial concerns, greater feelings of loneliness and a reduced sense of belonging. These findings were consistent across all domains of racial discrimination, indicating that racial discrimination in any setting can negatively impact mental health. Anti‐racist interventions which target the interconnected dimensions of racism are needed.

## 1 Introduction

### 1.1 Racism & Mental Health

The detrimental impact of racism on the mental health of ethnic minority people and Indigenous Peoples is well evidenced (Paradies et al. 2015). Racial discrimination at an interpersonal level (e.g., bullying and harassment), from both extreme experiences of racist hate crimes (e.g., violence and aggression) to discrimination in more covert forms (e.g., micro‐aggressions), contributes to poor mental health (Gómez 2015). There is compelling evidence that racial discrimination is related to both severe mental illness (SMI), such as psychosis (Lazaridou et al. 2023), and common mental disorders (CMD), such as depression and anxiety (Wallace et al. 2016).

Racial discrimination directly impacts physical and mental health, as these experiences, or fear of these experiences, provoke a stress response which strongly influences mental states (Dziurkowska and Wesolowski 2021), and chronically elevates cortisol levels (Adam et al. 2020). Elevated cortisol levels are considered one of the most notable biomarkers of depression and anxiety (Dziurkowska and Wesolowski 2021). Both actual experiences of racial discrimination and perceived risk of such experiences are a threat to one's identity and sense of security, as incidents of interpersonal racism are racially motivated attacks on a member of a community, not just an individual (Karlsen and Nazroo 2004). Therefore, learning about racial violence can have spillover effects on the mental health of members of ethnic minority groups (Bor et al. 2018).

**FIGURE 1:**
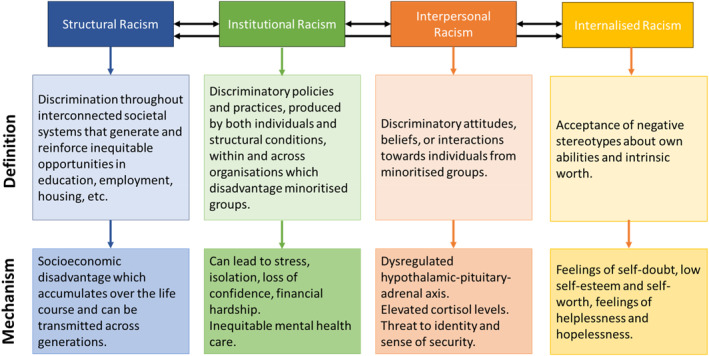
Definitions of racisms and the mechanisms by which they contribute to poor mental health.

Racism also occurs at structural and institutional levels (Figure 1). Structural racism is the embodiment of discrimination throughout interconnected societal systems that result in socioeconomic disadvantage through inequitable opportunities and access to resources (Bailey et al. 2017). Institutional racism occurs between structural and interpersonal levels, as institutional practices are produced by both individuals and structural conditions (Nazroo et al. 2020), resulting in discriminatory policies which disadvantage people from ethnic minority groups (Jones 2000). Socioeconomic disadvantage accumulates across the life‐course and can be transmitted across generations (Wallace et al. 2016), contributing to ethnic inequalities in physical and mental health outcomes (Adriaanse et al. 2014).

Though the association between racial discrimination and mental health is well evidenced, most studies have examined experiences of racial discrimination at any previous time or experienced recently. This impedes our understanding of the chronicity and accumulation of exposure to racism, and how this subsequently impacts mental health (Bécares et al. 2024). Few studies have captured how the types or domains of racist experiences (e.g., institutional racism in education or employment compared to racist assaults) may have differential impacts on mental health. Of the few studies that have captured exposure to racism at multiple time points and across multiple domains, there is evidence of a dose–response relationship (Harris et al. 2012; Wallace et al. 2016), whereby the size of the effect of racism on mental health increases with the number of times and domains that racism was experienced.

### 1.2 Impact of the COVID‐19 Pandemic

The COVID‐19 pandemic worsened mental health for many people in the United Kingdom (UK) (Pierce et al. 2020). Longitudinal data suggested that mental health worsened for certain ethnic minority groups, particularly mixed and Asian ethnic groups (Pierce et al. 2021). Recent data from the Evidence for Equality National Survey (EVENS), the largest cross‐sectional survey of ethnic minority people in the UK, identified greater levels of anxiety among people identifying as Arab, mixed White and Black Caribbean, any other mixed background, and any other Black background, compared to White British people, during the COVID‐19 pandemic (Irizar et al. 2024). The literature presented thus far outlines the association between racial discrimination and mental health among ethnic minority people. Data emerging from the COVID‐19 pandemic suggests that mental health may have worsened for certain ethnic minority groups, compared with the White majority. This paper seeks to understand how experiences of racial discrimination across the life course directly contribute to poor mental health among ethnic minority people, and how racial discrimination contributes to inequalities in the impact of the COVID‐19 pandemic, which in turn negatively affects mental health.

### 1.3 Theorised Pathways

It is now widely known that the COVID‐19 pandemic disproportionately affected people from ethnic minority groups, in terms of both health and economic outcomes, exacerbating existing inequalities (Platt and Warwick 2020; Irizar, Pan, et al. 2023). We theorise that experiences of racism across the life course contributed to the disproportionate health and economic impacts of COVID‐19. Structural and institutional racism contribute to longstanding socioeconomic disadvantage (Stopforth et al. 2022), with ethnic minority groups being more likely to be employed in insecure contracts without adequate sick pay, reducing the ability to self‐isolate, which increases the risk of infection and contributes to financial concerns (Nazroo and Bécares 2021). Additionally, socioeconomic inequalities drive the risk of infection through greater reliance on public transport, reduced access to open spaces, exposure to air pollution, and overcrowding (Platt and Warwick 2020; Hong et al. 2021). Moreover, population studies have demonstrated associations between both infection and financial insecurity with poor mental health (Ganson et al. 2021; Liu et al. 2021). Therefore, we hypothesise that experiences of racial discrimination, particularly in institutional settings, will be associated with greater odds of COVID‐19 infection and financial insecurity, which will in turn be associated with poor mental health.

**FIGURE 2:**
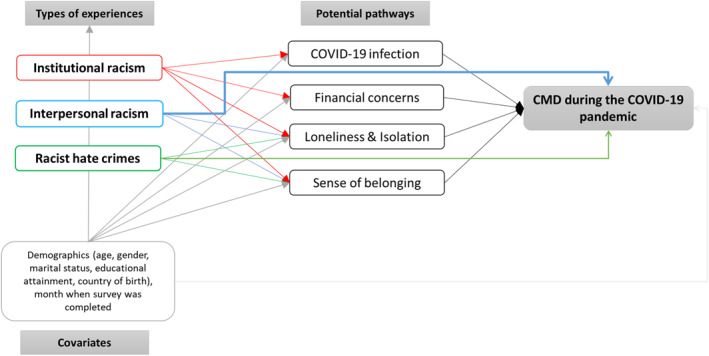
Conceptual model outlining the theorised pathways between institutional and interpersonal racism, and hate crimes, with CMD, through direct and indirect pathways.

Prior to the pandemic, evidence from the UK suggested that most ethnic minority groups experienced similar levels of CMD to White British people (with slightly higher estimates observed for Black people) (Ahmad et al. 2022). This is puzzling given the wide range of previously outlined social and economic risk factors for CMD. One explanation might be that the geographical concentration of ethnic minority populations (ethnic density) strengthens community belonging and social connections, enabling greater access to support and protecting against the negative effects of racism (Bécares et al. 2018). However, the COVID‐19 pandemic and associated restrictions may have damaged the sense of belonging in communities, contributing to increases in loneliness and isolation (Ernst et al. 2022). Moreover, the pandemic saw heightened experiences of racial discrimination, particularly towards Chinese people (Elias et al. 2021), which may have further undermined one's sense of belonging. Evidence suggests that racial discrimination is associated with loneliness and a reduced sense of belonging (Priest et al. 2017; Hussain and Jones 2021). We theorise that experiences of racial discrimination in all domains will contribute to greater loneliness and isolation, as well as a lower sense of community belonging—feelings which may have worsened during the COVID‐19 pandemic—which will be, in turn, associated with CMD. The theorised pathways are outlined in Figure 2.

We hypothesise a direct dose–response relationship between experiences of racism across the life course and the odds of reporting CMD during the COVID‐19 pandemic (theorising that racism is a stressor that directly impacts mental health), with the size of the effect increasing with cumulative exposure over time, in terms of both the number of times and domains in which racial discrimination has been encountered. It is anticipated that more recent experiences of racial discrimination will have stronger associations with CMD during the pandemic, and with feelings such as loneliness and belonging, compared to experiences of racial discrimination over 5–10 years ago (which may have resulted in negative emotional states at the time), with cumulative experiences over time having the strongest associations, through direct and indirect pathways. The type of racism experienced is hypothesised to contribute to CMD during the pandemic through different pathways, with hate crimes and interpersonal racism likely having a direct effect on CMD, whereas institutional racism likely has more indirect effects on CMD.

### 1.4 Research Objectives

This analysis aims to understand the associations between experiences of racial discrimination across the life course on the mental health of ethnic minority people, during the COVID‐19 pandemic. This work focuses both on when racial discrimination was experienced and the type of racial discrimination experienced, whilst also capturing cumulative experiences over time. Path analyses are used to identify how racial discrimination contributes to the disproportionate health and economic impacts of COVID‐19, social isolation, and a lower sense of community belonging, and the subsequent impacts on mental health through these pathways.

## 2 Methods

### 2.1 Study Population

Data for this secondary analysis were obtained from the Evidence for Equality National Survey (EVENS) (Finney et al. 2023, 2024), which aimed to recruit more people from ethnic minority groups than existing UK surveys, obtaining responses from people from 20 different ethnic minority groups, as well as White British people. For the purpose of this analysis, people only from ethnic minority groups, aged 18–60 years old, were included, as the majority of participants aged over 60 years old were White British and most White British respondents reported no experiences of racial discrimination. Respondents who identified as ‘any other White background’ were included, as 55% reported experiences of racial discrimination.

### 2.2 Data Collection

Data collection methods for this non‐probability survey are described in detail in the open access EVENS book (Finney et al. 2023). EVENS employed a responsive survey design, whereby the team monitored responses to the survey daily and used targeted recruitment strategies to achieve desired sample sizes for each ethnic group, by age, sex, and region (Shlomo et al. 2023). Data were collected between February and November 2021. To determine eligibility (aged 18 and over, living in England, Scotland, or Wales), respondents first completed an open‐link registration survey. If eligible, a unique link was provided to the main online survey. Snowball sampling was additionally used, whereby those who completed the survey received four links that could be passed on to family and friends. In addition, participants were able to complete the survey via telephone or face‐to‐face. The final sample of EVENS also included data from established web panels (Ipsos and Prolific), which were used to fulfil desired sample sizes. Data were collected in partnership with voluntary, community, faith and social enterprise (VCFSE) organisations serving ethnic minority populations, who supported the targeted recruitment strategies. Some ethnic minority groups that are typically under‐represented in probability‐based surveys (e.g., people from Roma backgrounds) were oversampled to ensure a minimum target sample. After completing the survey, participants were given a £10 voucher as a ‘thank you’.

### 2.3 Sampling Weights

Statistical adjustment weights were created by data custodians, due to the non‐probability sampling approach, to account for selection bias and coverage bias, and to produce generalisable results (Shlomo et al. 2023). The non‐probability sample was integrated with a probability reference sample, to estimate propensity scores, which were then used to calculate pseudo‐design weights. Following this, the pseudo‐design weights were calibrated to population benchmarks (obtained from the 2021 Census for England and Wales and the ETHPOP Database for Scotland). The calibrated weights were then scaled so that the sample matched the population benchmarks in terms of age, sex, region, and ethnic group.

### 2.4 Exposure

The measure of racial discrimination included in EVENS is an abbreviated and modified measure of life course experiences of racial discrimination developed in collaboration with colleagues in the United States, New Zealand, and the UK (Bécares et al. 2024). Participants were asked if they had experienced discrimination for reasons to do with their ethnicity, race, colour, or religion across 10 domains and four time periods (with additional options for ‘don't know’ and ‘this hasn't happened to me’). For each domain, participants responded with yes or no to each time period. There were a total of 60 items measuring experiences of racism in various domains and time periods (Supporting Information Table S1). Responses were coded as a binary variable (1/0), with positive responses to each time period coded as 1. Responses of ‘don't know’ were coded as missing and responses of ‘this hasn't happened to me’ were coded as 0.

The 10 domains were then reduced to three theorised domains: hate crimes (having property deliberately damaged and being physically attacked), interpersonal racial discrimination (being insulted, treated unfairly in public, treated unfairly by friends/family/partner and neighbours made life difficult for you), and experiences of racial discrimination in institutional settings (treated unfairly in education, treated unfairly in work, treated unfairly by police and treated unfairly when seeking housing). Interpersonal racial discrimination typically covers a range of incidents, from ‘microaggressions’ to hate crimes, yet we have included hate crimes as a separate domain as, in the UK, this is defined as a criminal offence that is typically motivated by prejudice on the basis of perceived membership to a certain social group (Crown Prosecution Service 2022).

To comprehensively capture the impact of experiences of racial discrimination over time on mental health, several exposure variables were created (Table S1). The first exposure variable measures cumulative experiences of racial discrimination across time points and domains. The second exposure variable captures differences in the timing of experiences of racial discrimination (recent experiences only, past experiences only and chronic experiences of racial discrimination). The final set of exposure variables replicate the previous variable but separately for each of the three theorised domains (hate crimes, interpersonal racism and racial discrimination in institutional settings), capturing differences between domains.

### 2.5 Outcome

The outcome of interest is self‐reported CMD, that is, depression and anxiety. Depression was assessed using the centre for epidemiologic studies depression scale (CES‐D‐8), which contains eight items measuring symptoms of depression during the past week (Radloff 1977). Yes or no responses were provided for each item (scores range from 0 to 8; items 4 and 6 were reverse coded), with a score of four or more indicating probable depression (Zaninotto et al. 2013). Anxiety was assessed using the generalised anxiety disorder‐7 (GAD‐7), which includes seven items measuring symptoms of anxiety during the past 2 weeks (Kroenke et al. 2007). Responses were given on a Likert scale, ranging from ‘not at all’ (0) to ‘nearly every day’ (3). Scores range from 0 to 21, with a validated cut‐off of 10 or more indicating moderate to severe anxiety (Kroenke et al. 2007). A binary variable was created, reflecting caseness for depression and/or anxiety (CMD) versus neither depression nor anxiety.

### 2.6 Potential Pathways

#### 2.6.1 Health Impacts of the COVID‐19 Pandemic

Participants were asked if they had ever been tested for COVID‐19 (yes/no) and for those who had been tested, if they had ever received a positive result for COVID‐19 (yes/no/prefer not to say). These items were combined to determine previous infection.

#### 2.6.2 Economic Precarity

When completing the survey, participants were asked about changes in current household income compared to before the COVID‐19 pandemic began. Responses were coded as higher (‘much higher’ and ‘a little higher’ (1)), about the same (2) and lower (‘much lower’ and ‘a little lower’ (3)). Participants were also asked how worried, if at all, they were about their future financial security: not at all worried (0), somewhat worried (1), very/extremely worried (2) and prefer not to say (3).

#### 2.6.3 Loneliness

Four items measured loneliness. The first three items were taken from the validated UCLA loneliness scale (Hughes et al. 2004), asking respondents how often they lack companionship, feel left out and feel isolated from others. An additional item directly measured loneliness (GOV.UK 2023), asking how often participants feel lonely. Response options included ‘hardly ever or never’ (0), ‘some of the time’ (1), ‘often’ (2). Responses to the four items were summed to create a continuous measure of loneliness, ranging from 0 to 8. Additionally, participants were asked whether their feelings of loneliness and isolation had changed since the start of the COVID‐19 pandemic (grouping ‘decreased’ and ‘stopped’ (1), versus ‘stayed the same’ (2) and ‘increased’ (3)).

#### 2.6.4 Belonging

Respondents were asked how strongly they feel they belong to their local area, that is, within a 15‐min walk from their home (very/fairly strongly (0) and not very/not at all strongly (1)) (Ipsos MORI and TNS‐BMRB 2010). Additionally, they were asked if they thought their sense of belonging to the local community has changed since the COVID‐19 pandemic began (MOPAC 2022), with responses coded as increased (grouping ‘increased a lot’ and ‘increased a little’ (1)), no change (2) and decreased (grouping ‘decreased a little’ and ‘decreased a lot’ (3)).

### 2.7 Covariates

The following variables were included as covariates: age (continuous and as a squared term), sex (male/female), educational attainment (higher education, A‐level or equivalent or vocational qualification, GCSE or below and other), marital status (married or civil partnership, never married, divorced or separated or civil partnership dissolved and widowed or surviving partner from civil partnership), country of birth (UK‐born and foreign‐born) and month when the survey was completed.

### 2.8 Data Analysis

Descriptive statistics (frequencies and percentages, means and standard deviations) were used to summarise the sample, stratified by CMD caseness, for the demographic, pathway, and exposure variables (Tables S2–S4). Regression models, controlling for covariates, were first conducted to estimate the independent associations between (i) each of the exposure variables and CMD during the pandemic (logistic regression), (ii) each of the exposure variables and the potential pathway variables (multinomial/linear regressions) and (iii) the potential pathway variables and CMD during the pandemic (logistic regression).

**FIGURE 3:**
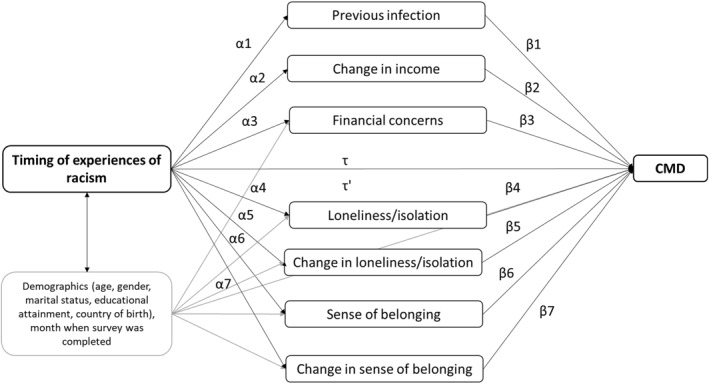
Theorised path analysis model between timing of experiences of racial discrimination (no reported experiences, past experiences only, recent experiences only and both past and recent experiences), potential pathways and CMD.

Structural equation modelling (SEM) is widely used to explore complex relationships between variables in epidemiological studies and performs well for resolving the endogeneity problem (i.e., when the explanatory variable is correlated with variation, or ‘error’, in the outcome variable) (Hult et al. 2018). SEM enables an investigation of direct, indirect and total effects between exogenous and endogenous variables. However, in SEM, all responses are assumed to be continuous. Therefore, generalised structural equation modelling (GSEM) can be used to avoid biases attributable to the use of binary, ordinal and nominal variables (Zhang and Zhang 2018). Timing of experiences of racial discrimination was chosen as the primary exposure for the GSEM, as it showed more meaningful variation than the measure of cumulative exposure to racial discrimination, and a tetrachoric correlation matrix identified that the domains of racism were highly correlated (correlation matrices are presented in Supporting Information Table S5). First, variables were checked to ensure they met assumptions of multicollinearity (variance inflation factor below 5) (Kim 2019). Then, path analysis using GSEM was applied to estimate the associations between racial discrimination and the potential pathways (*Path* α); the associations between the potential pathways and CMD *(Path β)*; the association between racial discrimination and CMD, the total effect *(Path τ)* and the association between racial discrimination and CMD adjusted for the pathways, the direct effect *(Path τ′)*. See Figure 3. Indirect effects were calculated as the product of the coefficients αβ.

Analyses were performed using STATA SE 15 and were weighted to account for selection bias and coverage bias, due to the non‐probability nature of the sample, using the *svy* command. Applying these weights means that the sample can be assumed to be representative of the UK general population. Direct, indirect and total effects were obtained using the *nlcom* command and reported as adjusted odds ratios (exponentiating coefficients to ease interpretation) with 95% confidence intervals.

### 2.9 Missing Data

Missing data (including ‘prefer not to say” and ‘don't know’ responses) were minimal, that is, less than 5% for each demographic, pathway and exposure variable (Tables S2–S4). To maximise the data available, the sample size was allowed to vary for each path in the GSEM. For the outcome, 13.24% of participants responded with ‘prefer not to say’ for at least one item of the CES‐D‐8 and 7.02% for at least one item of the GAD‐7 (coded as missing as this cannot be used to create total score). For participants with complete data on at least 80% of items person mean imputation, averaging responses to available items, was conducted to compute total scale scores (*N* = 8525 for depression, *N* = 8759 for anxiety, *N* = 8897 for combined CMD). As a sensitivity analysis, complete case analyses using pairwise deletion are presented for the GSEM in Supporting Information Table S11 (*N* = 7069 for depression, *N* = 8393 for anxiety, *N* = 8672 for combined CMD).

## 3 Results

### 3.1 Sample Characteristics

The demographic characteristics of the sample (*N* = 8,897, 52% female) and the proportion of participants from each ethnic minority group are presented in Supporting Information (Table S2). In total, 45.4% of the sample met the criteria for either depression or anxiety. Only 12% had tested positive for COVID‐19 infection prior to completing the survey, 28% reported that their household income was lower than before the pandemic and 23% were very or extremely worried about their future financial situation and 37% reported an increase in loneliness and isolation, though only 14% reported a reduction in feelings of belonging (Table S3). Less than 30% of the sample reported no experiences of racial discrimination, with 16% reporting past experiences of racial discrimination but no recent experiences, 25% reporting recent experiences of racial discrimination without past experiences and 30% reported both past and recent experiences of racial discrimination (Table S4).

### 3.2 Regression Analyses

We found evidence of a dose–response relationship between experiences of racial discrimination over time and the odds of reporting CMD during the COVID‐19 pandemic (Table 1). Compared with no reported experiences of racial discrimination, the odds of reporting CMD became greater with increasing number of domains and time‐points that racial discrimination was experienced. Those who had experienced racial discrimination in all 3 domains at 3 or 4 time points were over 4 times more likely to report CMD compared with those who had not experienced racial discrimination (OR = 4.65, 95%CI: 3.59–6.03), and those who had experienced racial discrimination in 2 domains at 1 or 2 time points were twice as likely (OR = 2.01, 95%CI: 1.59–2.54). The association was not as strong for those who had experienced racial discrimination in 1 domain at 1 or 2 time points (OR = 1.31, 95%CI: 1.02–1.68).

**TABLE 1:** Independent associations between each of the exposure variables and CMD during the pandemic.

Variable	OR (95% CI) *N* = 8897	aOR (95% CI) *N* = 8888
Cumulative experiences of racism		
No experiences of racism	1.00	1.00
Racism in 1 domain at 1 or 2 time points	1.29 (1.01–1.66)*	1.31 (1.02–1.68)*
Racism in 1 domain at 3 or 4 time points	2.80 (1.14–6.86)*	2.75 (1.18–6.44)*
Racism in 2 domains at 1 or 2 time points	1.91 (1.51–2.43)***	2.01 (1.59–2.54)***
Racism in 2 domains at 3 or 4 time points	2.41 (1.77–3.28)***	2.35 (1.73–3.19)***
Racism in 3 domains at 1 or 2 time points	3.00 (2.29–3.93)***	3.19 (2.40–4.23)***
Racism in 3 domains at 3 or 4 time points	3.42 (3.36–5.56)***	4.65 (3.59–6.03)***
Timing of experiences of racism		
No experiences of racism	1.00	1.00
Past experiences but not recent	1.38 (1.07–1.77)*	1.50 (1.16–1.93)**
Recent experiences but not past	2.08 (1.66–2.61)***	2.01 (1.60–2.52)***
Past and recent experiences	2.99 (2.42–3.69)***	3.02 (2.44–3.74)***
Timing of experiences of hate crimes		
No experiences of racism	1.00	1.00
Past experiences but not recent	1.71 (1.33–2.18)***	1.87 (1.46–2.40)***
Recent experiences but not past	2.70 (2.20–3.31)***	2.70 (2.17–3.36)***
Past and recent experiences	3.06 (1.65–5.68)***	3.30 (1.73–6.26)***
Timing of experiences of interpersonal racism		
No experiences of racism	1.00	1.00
Past experiences but not recent	1.59 (1.26–2.02)***	1.69 (1.34–2.15)***
Recent experiences but not past	2.47 (2.04–2.99)***	2.39 (1.97–2.90)***
Past and recent experiences	3.05 (2.25–2.25)***	3.03 (2.20–4.16)***
Timing of experiences of institutional racism		
No experiences of racism	1.00	1.00
Past experiences but not recent	1.67 (1.34–2.08)***	1.78 (1.42–2.22)***
Recent experiences but not past	2.72 (2.27–3.25)***	2.63 (2.19–3.16)***
Past and recent experiences	2.96 (2.08–4.22)***	2.97 (2.08–4.24)***

*Note:* Unadjusted and adjusted odds ratios (OR) are presented, with 95% Confidence Intervals (CIs). Analyses are weighted to account for selection bias and coverage bias. Adjusted analyses control for age, age squared, sex, educational attainment, marital status, country of birth, and month when survey was completed.

*p < 0.05, **p < 0.01, ***p < 0.001.

As hypothesised, recent experiences of racial discrimination without past experiences of racial discrimination were associated with twice the odds of reporting CMD (95%CI: 1.60–2.52), compared with no reported experiences of racial discrimination, whereas past experiences without recent experiences were associated with 1.5 times the odds (95%CI: 1.16–1.92) and chronic experiences over time (both past and recent experiences of racial discrimination) were associated with 3 times the odds (95%CI: 2.44–3.74). When exploring associations across the different domains of racial discrimination (hate crimes, interpersonal and in institutional settings) with CMD, there were minimal differences (see Table S5 for correlation matrix showing that all domains were strongly correlated).

The regression models showing the associations between each of the exposure variables and the potential pathway variables are reported in Supporting Information (Tables S6–S9). The associations between the potential pathway variables and CMD are reported in Supporting Information Table S10.

### 3.3 Path Analyses

Table 2 shows the results of the path analysis model examining the associations between timing of experiences of racial discrimination and CMD, via SARS‐CoV‐2 infection, change in income, financial concerns, loneliness and isolation, changes in loneliness and isolation, sense of belonging and change in sense of belonging. The columns indicate the different categories of the exposure variable and the rows indicate the different paths in the model.

**TABLE 2:** Final model of the path analysis estimating the relationship between experiences of racism (reference = no experiences of racism) and CMD (reference = no CMD), through potential pathways (*N* = 8421).

	CMD		Past experiences		Recent experiences		Past & Recent	
	aOR	95% CI	aOR	95% CI	aOR	95% CI	aOR	95% CI
*Path α*								
Racism → previous infection *α1*			1.00		1.00		1.00	
Yes			1.04	0.72 to 1.50	1.44*	1.04 to 1.98	1.28	0.94 to 1.73
Racism → change in income *α2*			1.00		1.00		1.00	
About the same			0.89	0.64 to 1.23	0.66**	0.49 to 0.88	0.81	0.62 to 1.06
Lower			1.11	0.77 to 1.60	1.10	0.80 to 1.51	1.23	0.91 to 1.65
Racism → financial concerns *α3*			1.00		1.00		1.00	
Somewhat concerned			1.28	0.94 to 1.75	1.53**	1.17 to 2.00	1.96***	1.50 to 2.56
Very/extremely concerned			1.55*	1.04 to 2.32	2.54***	1.77 to 3.65	3.14***	2.22 to 4.43
Racism → loneliness *α4 (βeta coefficient)*			0.69***	0.42 to 0.97	0.94***	0.70 to 1.19	1.29***	1.06 to 1.52
Racism → change in loneliness *α5*			1.00		1.00		1.00	
Stayed the same			0.87	0.59 to 1.28	0.58**	0.41 to 0.80	0.57**	0.42 to 0.79
Increased			1.21	0.81 to 1.80	1.04	0.74 to 1.45	1.23	0.89 to 1.69
Racism → belonging *α6*			1.00		1.00		1.00	
Not very/not at all			1.05	0.76 to 1.45	1.19	0.92 to 1.54	1.42**	1.12 to 1.81
Racism → change in belonging *α7*			1.00		1.00		1.00	
Increased			0.97	0.73 to 1.29	1.27	1.00 to 1.62	1.36**	1.08 to 1.71
Decreased			1.07	0.71 to 1.61	2.16***	1.47 to 3.18	2.12***	1.49 to 3.01
*Path β*								
Previous infection → CMD *β1*	1.00							
Yes	1.24	0.91 to 1.68						
Change in income → CMD *β2*	1.00							
About the same	1.08	0.84 to 1.38						
Lower	1.06	0.80 to 1.41						
Financial concerns → CMD *β3*	1.00							
Somewhat concerned	1.81***	1.40 to 2.33						
Very/extremely concerned	4.03***	2.89 to 5.70						
Loneliness → CMD *β4*	1.54***	1.46 to 1.62						
Change in loneliness → CMD *β5*	1.00							
Stayed the same	0.87	0.66 to 1.16						
Increased	1.67***	1.25 to 2.22						
Belonging → CMD *β6*	1.00							
Not very/not at all	1.44**	1.14 to 1.82						
Change in belonging → CMD *β7*	1.00							
Increased	1.47**	1.18 to 1.82						
Decreased	1.47*	1.06 to 2.04						
Path α1*β1								
Racism → CMD (indirect through previous infection)			1.00		1.00		1.00	
Yes			1.09	0.75 to 1.44	1.57*	1.05 to 2.09	1.85*	1.30 to 2.40
Path α2*β2								
Racism → CMD (indirect through change in income)			1.00		1.00		1.00	
About the same			1.08	0.74 to 1.41	1.40	0.96 to 1.85	1.73*	1.24 to 2.21
Lower			1.09	0.75 to 1.43	1.46*	1.02 to 1.89	1.77*	1.27 to 2.28
Path α3*β3								
Racism → CMD (indirect through financial concerns)			1.00		1.00		1.00	
Somewhat concerned			1.26	0.80 to 1.72	1.87*	1.22 to 2.51	2.61*	1.66 to 3.56
Very/extremely concerned			2.01	0.69 to 3.32	5.33*	1.85 to 8.80	8.63*	2.75 to 14.51
Path α4*β4								
Racism → CMD (indirect through loneliness)			1.46	0.97 to 1.95	2.18*	1.50 to 2.86	3.06*	2.17 to 3.94
Path α5*β5								
Racism → CMD (indirect through change in loneliness)			1.00		1.00		1.00	
Stayed the same			1.11	0.76 to 1.45	1.56*	1.03 to 2.09	1.89*	1.28 to 2.50
Increased			1.20	0.73 to 1.66	1.48	0.94 to 2.01	1.95*	1.31 to 2.59
Path α6*β6								
Racism → CMD (indirect through belonging)			1.00		1.00		1.00	
Not very/not at all			1.10	0.74 to 1.47	1.54*	1.06 to 2.03	1.99*	1.38 to 2.60
Path α7*β7								
Racism → CMD (indirect through change in belonging)			1.00		1.00		1.00	
Increased			1.07	0.72 to 1.43	1.59*	1.08 to 2.10	1.97*	1.37 to 2.57
Decreased			1.11	0.72 to 1.50	1.95*	1.11 to 2.79	2.34*	1.41 to 3.26
*Path τ*								
Total effects			1.50**	1.16 to 1.94	2.11***	1.67 to 2.67	2.91***	2.33 to 3.65
*Path τ′*								
Direct effects			1.09	0.80 to 1.48	1.45*	1.08 to 1.95	1.75***	1.33 to 2.31

*Note:* Analyses are weighted to account for selection bias and coverage bias. N for each response: CMD = 7886; timing of experiences of racism = 8421; previous infection = 8394; change in household income = 7860; financial concerns = 8265; loneliness and isolation = 8421; change in loneliness and isolation = 7860; belonging = 8246; change in belonging = 8203. Reference groups: previous infection = no; change in household income = higher; financial concerns = not at all worried; changes in loneliness = decreased/stopped; belonging = very/fairly strongly; change in belonging = no change. For indirect effects, statistical significance was determined as confidence intervals without the value of 1.

*p < 0.05, **p < 0.01, ***p < 0.001.

Recent experiences of racial discrimination were marginally associated with previous infection (OR = 1.44, 95%CI: 1.04–1.98) and both recent and cumulative experiences of racial discrimination were indirectly associated with CMD via SARS‐CoV‐2 infection (recent OR = 1.57, 95%CI: 1.05 to 2.09; cumulative OR = 1.85, 95%CI: 1.30–2.40). Recent and cumulative experiences of racial discrimination (but not past experiences) were associated with greater odds of reporting financial concerns and indirectly associated with greater odds of CMD via financial concerns. Experiences of racial discrimination were not associated with lower income, nor was lower income associated with CMD, yet recent and past experiences of racial discrimination were indirectly associated with CMD via lower income (recent OR = 1.46, 95%CI: 1.02 to 1.89; cumulative OR = 1.77, 95%CI: 1.27–2.28).

Experiences of racial discrimination at any time point, compared with no reported experiences of racial discrimination, were strongly associated with greater feelings of loneliness and isolation, and recent/chronic experiences of racial discrimination (but not past) were indirectly associated with CMD through greater feelings of loneliness and isolation. Experiences of racial discrimination at any time point were not associated with increases in loneliness and isolation, but cumulative experiences of racial discrimination were indirectly associated with CMD through increased feelings of loneliness and isolation (OR = 1.95, 95%CI: 1.31–2.59).

Cumulative experiences of racial discrimination were associated with a low sense of belonging, and both recent and cumulative experiences of racial discrimination were associated with decreased feelings of belonging during the pandemic. Recent and cumulative experiences of racial discrimination were also indirectly associated with CMD via a low sense of belonging and decreased feelings of belonging during the pandemic.

The total effects of experiences of racial discrimination on CMD (without including potential pathways in the model) show that, compared with no reported experiences of racial discrimination, past experiences of racial discrimination were associated with 1.50 times the odds of reporting CMD (95%CI: 1.16–1.94), recent experiences of racial discrimination were associated with 2.11 times the odds (95%CI: 1.67–2.67) and both past and recent experiences were associated with 2.91 times the odds of reporting CMD (95%CI: 2.33–3.65). When the potential pathways were included (direct effects), the effect sizes attenuated and past experiences of racial discrimination were no longer associated with CMD (OR = 1.09, 95%CI: 0.80–1.48). However, recent experiences and cumulative experiences of racial discrimination were still associated with greater odds of reporting CMD (recent OR = 1.45, 95%CI: 1.08 to 1.95; cumulative OR = 1.75, 95%CI: 1.33–2.28).

These findings were consistent in the complete case analysis (Table S11) and mostly harmonious across each of the domains of racial discrimination (Table S12–S14).

## 4 Discussion

We took a theoretically informed approach to identify the associations between experiences of racial discrimination over time, capturing the accumulation and chronicity of these experiences, with mental health, through direct and indirect pathways. In line with our conceptual model and previous literature (Harris et al. 2012; Wallace et al. 2016), we found clear evidence of a direct dose–response relationship between experiences of racial discrimination and self‐reported CMD for ethnic minority people. This is concerning given that over 70% of participants reported experiencing racial discrimination at least once and 30% experienced chronic racial discrimination across their life. Additionally, we identified that recent experiences of racial discrimination are more strongly associated with CMD than past experiences of racial discrimination, in line with wider mental health literature whereby recent stressors have a stronger impact on mental health (Naicker et al. 2021). Cumulative experiences (both past and recent) had the strongest association with CMD, as theorised. These findings were consistent across all domains of racial discrimination, opposing our hypothesis that racial discrimination in institutional settings would only have indirect effects on CMD, indicating that racial discrimination in any form can have a direct negative impact on mental health. Racisms exist as historically and politically determined structures of domination, thus, experiencing racism in any domain has psychological impacts through reinforced marginalisation, disempowerment and threats to ontological security (Nazroo et al. 2020).

We show that racial discrimination is directly associated with mental health and this is supported by existing evidence, demonstrating clear neurobiological and physiological pathways (Paradies et al. 2015). When looking at the indirect effects of racial discrimination, we found that recent and chronic experiences of racial discrimination were associated with poor mental health through financial insecurity, aligning with our conceptual model and building on US literature (Despard et al. 2023). Racism results in inequitable access to economic resources, as demonstrated by persisting ethnic inequalities in education, housing, employment and income. For example, unemployment levels have been consistently higher for Black Caribbean, Black African, Pakistani and Bangladeshi people, compared with White British people, for over 2 decades (Kapadia et al. 2015). Financial insecurity has been found to be causally associated with poor mental health in the wider UK general population both before (Kopasker et al. 2018) and during the pandemic (Cheng et al. 2021). We now show that racism is associated with economic precarity, and given previous longitudinal evidence on the causal mechanisms, it is plausible that this then contributes to poor mental health among ethnic minority people.

We theorised that experiences of racial discrimination at any time would be associated with greater feelings of loneliness and a lower sense of belonging, worsened feelings since the pandemic began and indirectly associated with CMD through these pathways. The findings mostly support this, though past experiences of racial discrimination were only associated with greater feelings of loneliness and were not indirectly associated with CMD through these pathways. Longitudinal evidence supports the causal and unidirectional association between racial discrimination and subsequent feelings of loneliness (Priest et al. 2017). The present evidence builds on this work, now showing that racial discrimination indirectly impacts mental health through greater feelings of loneliness. Few existing studies had examined the relationship between racial discrimination and belonging among ethnic minority people living in Britain, with our findings demonstrating a clear association between racial discrimination and a low sense of belonging, which in turn was associated with poor mental health.

Based on population studies (Liu et al. 2021) and prior theoretical models (Irizar, Kapadia, et al. 2023), we hypothesised that racial discrimination would be associated with SARS‐CoV‐2 infection, which would then be associated with poor mental health. Contrarily, racial discrimination was not associated with previous infection nor was infection associated with CMD, yet recent and chronic experiences of racial discrimination were indirectly associated with CMD through greater odds of infection. There may be other factors which confound the relationship between racism and infection and between infection and mental health (e.g., relating to socioeconomic position or neighbourhood infrastructure). The theorised model enabled an exploration into various direct and indirect pathways between racial discrimination and mental health. However, potentially protective factors were not examined, and future research could investigate the roles of ethnic identity, ethnic density or religiosity, which could possibly protect against the adverse effects of racism, by strengthening social support, social identities, and resilience (Bécares et al. 2018).

### 4.1 Implications

These findings have important implications for policies and interventions which seek to improve the lives and wellbeing of ethnic minority people living in the UK. Interventions targeted at improving the mental health of ethnic minority people would be beneficial, especially as mental health treatment receipt is much lower for ethnic minority groups and this inequality has widened over time (Ahmad et al. 2022). In order to address inequalities in mental health care, services must reduce barriers to accessing equitable care, ensure early intervention, and tackle the societal drivers of poor mental health by centring care on racial trauma (Runnymede Trust 2023). However, these interventions would only address the consequence of racism and not the root cause. Anti‐racist interventions which target the interconnected dimensions of racism are needed, particularly as we find evidence to suggest that recent experiences of racial discrimination have a stronger effect on mental health than experiences of racial discrimination over 5 years ago, emphasising the need to take a preventative approach to reduce the risk of worsened mental health.

### 4.2 Strengths and Limitations

EVENS includes a larger sample of people from ethnic minority groups than any other UK survey. The inclusion of comprehensive measures of racial discrimination enables an in‐depth exploration of the chronicity and accumulation of these experiences and the impact they have on the lives of ethnic minority people (Bécares et al. 2024). Nevertheless, there are some limitations of this analysis. When analysing direct and indirect effects, it is assumed that there are no unmeasured confounders in the exposure–outcome association, mediator–outcome association or exposure–mediator association (VanderWeele 2010). This also requires an assumption of temporal ordering whereby the exposure precedes the outcome, mediators precede the outcome, and the exposure precedes mediators (VanderWeele 2010). Despite the cross‐sectional nature of the data, most of the measures can be used in a way that assumes temporal ordering and the direction of causality between racial discrimination and the hypothesised pathways are informed by theory and prior longitudinal evidence. However, it is possible that there are additional confounders that were not included (e.g., other stressful life events or adverse experiences) or that poor mental health precedes feelings of loneliness, reduced sense of belonging and financial concerns (e.g., unable to work due to poor mental health), particularly, as we were unable to control for prior CMD in the models. Thus, longitudinal analyses are required to confirm the present findings. Finally, though it is beyond the scope of this paper, it is plausible that the pathways between racial discrimination and mental health differ across ethnic groups, particularly, as previous research within this sample found differences in experiences of racism across ethnic groups (Bécares et al. 2024).

## 5 Conclusions

We found a direct dose–response relationship between experiences of racial discrimination and mental health during the COVID‐19 pandemic, supporting existing evidence. We now identify that recent experiences of racial discrimination have a stronger effect on mental health compared to experiences over 5 years ago. Guided by theory, we also show that racial discrimination indirectly impacts mental health through SARS‐CoV‐2 infection, financial insecurity, feelings of loneliness and a low sense of belonging. The pandemic exposed and exacerbated existing inequalities in physical and mental health, addressing the fundamental drivers of these inequalities is imperative to ensure equitable post‐pandemic recovery, particularly in the context of the current cost of living crisis as people from ethnic minority groups are disproportionately disadvantaged (Munro et al. 2022).

## Author Contributions

**Patricia Irizar:** conceptualization, formal analysis, funding acquisition, investigation, methodology, project administration, validation, visualization, writing – original draft preparation, writing – review and editing. **Dharmi Kapadia:** conceptualization, data curation, investigation, methodology, project administration, supervision, validation, visualization, writing – review and editing. **Harry Taylor:** conceptualization, data curation, methodology, validation, visualization, writing – review and editing. **Gertrude Wafula:** conceptualization, investigation, supervision, validation, visualization, writing – review and editing. **Charles Kwaku–Odoi:** conceptualization, investigation, methodology, supervision, validation, visualization, writing – review and editing. **Laia Becares:** conceptualization, data curation, investigation, methodology, supervision, project administration, supervision, validation, visualization, writing – review and editing. **Srinivasa Vittal Katikireddi:** conceptualization, investigation, methodology, project administration, supervision, validation, visualization, writing – review and editing.

## Ethics Statement

The University of Manchester's Research Ethics Committee approved the EVENS survey. Additional ethical approval was not required for this secondary analysis.

## Conflicts of Interest

SVK was co‐chair of the Scottish Government’s Expert Reference Group on Ethnicity and COVID‐19 and a member of the UK Scientific Advisory Group on Emergencies (SAGE) subgroup on ethnicity. All other authors declare no conflicts of interest.

## Supporting information

Supporting Information S1

## Data Availability

Data from the Evidence for Equality National Survey (EVENS) is available through the UK Data Service (10.5255/UKDA‐SN‐9116‐1).
