# Supplementary material for: Associations Between Experiences of Racial Discrimination Across the Life Course and Mental Health: Exploring Direct and Indirect Pathways
Source: Sociol Health Illn. 2025 Apr 9;47(4):e70023. doi: 10.1111/1467-9566.70023 (PMC11980026; doi:10.1111/1467-9566.70023)
Supplement: Supplementary file 1 — Supporting Information S1 [file SHIL-47-0-s001.docx]

**Supplementary Materials**

Contents

[**Table S1.** Description of exposure variables. 2](#_Toc170117946)

[**Table S2.** Demographic characteristics of sample (N = 8,897). Weighted percentages are shown. 3](#_Toc170117947)

[**Table S3.** Descriptive statistics for experiences during the pandemic. Percentages are weighted. 4](#_Toc170117948)

[**Table S4.** Descriptive statistics for experiences of racism across the life course. Percentages are weighted. 5](#_Toc170117949)

[**Table S5.** Tetrachoric correlation matrix for each domain of racism (yes at any time point vs no at all time points): hate crimes, interpersonal racism, institutional racism (N = 8,796). 6](#_Toc170117950)

[**Table S6.** Independent associations between each of the exposure variables and the health impacts of the pandemic. Weighted and adjusted multinomial odds ratios (MOR) are presented, with 95% Confidence Intervals (CIs) (N = 9,019). 7](#_Toc170117951)

[**Table S7.** Independent associations between each of the exposure variables and the economic impacts of the pandemic. Weighted and adjusted multinomial odds ratios (MOR) are presented, with 95% Confidence Intervals (CIs) (N = 9,019). 9](#_Toc170117952)

[**Table S8.** Independent associations between each of the exposure variables and loneliness. Beta values (β) and adjusted multinomial odds ratios (MOR) are presented, with 95% Confidence Intervals (CIs) (N = 9,019). Analyses are weighted. 10](#_Toc170117953)

[**Table S9.** Independent associations between each of the exposure variables and belonging. Weighted and adjusted multinomial odds ratios (MOR) are presented, with 95% Confidence Intervals (CIs) (N = 8,888). 11](#_Toc170117954)

[**Table S10.** Independent associations between each of the potential pathway variables and CMD during the pandemic. Unadjusted and adjusted odds ratios (OR) are presented, with 95% Confidence Intervals (CIs). Analyses are weighted to account for selection bias and coverage bias. 13](#_Toc170117955)

[**Table S11.** Complete case path analysis estimating the relationship between timing of experiences of racism and CMD, through potential pathways (N = 8,421). Analyses are weighted to account for selection bias and coverage bias. 14](#_Toc170117956)

[**Table S12.** Path analysis estimating the relationship between experiences of hate crimes and CMD, through potential pathways (N = 8,421). Analyses are weighted to account for selection bias and coverage bias. 17](#_Toc170117957)

[**Table S13.** Path analysis estimating the relationship between experiences of interpersonal racism and CMD, through potential pathways (N = 8,391). Analyses are weighted to account for selection bias and coverage bias. 20](#_Toc170117958)

[**Table S14.** Path analysis estimating the relationship between experiences of institutional racism and CMD, through potential pathways (N = 8,332). Analyses are weighted to account for selection bias and coverage bias. 23](#_Toc170117959)

#

# **Table S1.** Description of exposure variables.

| **Measures of racial discrimination** | |  |
| --- | --- | --- |
| EVENS items | | *Yes/No to each item:* |
|  | 1. Has anyone insulted you for reasons to do with your ethnicity, race, colour or religion? | In the past year |
|  | 1. Has anyone deliberately damaged any property that belonged to you for reasons to do with your ethnicity, race, colour or religion? | In the past 5 years |
|  | 1. Has anyone physically attacked you for reasons to do with your ethnicity, race, colour or religion? | Within the last 10 years |
|  | 1. In education, have you ever been treated unfairly because of your ethnicity, race, colour or religion? | Over 10 years ago |
|  | 1. In your job, have you ever been treated unfairly because of your ethnicity, race, colour or religion? | Don’t know |
|  | 1. When you have been out in public, have you ever been treated unfairly because of your ethnicity, race, colour or religion? | This has not happened to me |
|  | 1. Have you ever been treated unfairly because of your ethnicity, race, colour or religion by the police? |  |
|  | 1. When seeking housing, have you ever been treated unfairly because of your ethnicity, race, colour or religion? |  |
|  | 1. Have any neighbours ever made life difficult for you or your family because of your ethnicity, race, colour or religion? |  |
|  | 1. Have you ever been treated unfairly by your friends, family, or partner because of your ethnicity, race, colour or religion? |  |
| Cumulative experiences of racism | | 7-level categorical variable |
|  | No experiences of racism |  |
|  | Racism in 1 domain at 1 or 2 time points |  |
|  | Racism in 1 domain at 3 or 4 time points |  |
|  | Racism in 2 domains at 1 or 2 time points |  |
|  | Racism in 2 domains at 3 or 4 time points |  |
|  | Racism in 3 domains at 1 or 2 time points |  |
|  | Racism in 3 domains at 3 or 4 time points |  |
| Timing of experiences of racism | | 4-level categorical variable |
|  | No experiences of racism |  |
|  | Racism over 5 years ago (but not within the past 5 years) |  |
|  | Racism within the past 5 years (but not over 5 years ago) |  |
|  | Both past and recent experiences of racism |  |
| Timing of experiences of hate crimes | | 4-level categorical variable |
|  | No experiences of hate crimes |  |
|  | Hate crime over 5 years ago (but not within the past 5 years) |  |
|  | Hate crime within the past 5 years (but not over 5 years ago) |  |
|  | Both past and recent experiences of hate crimes |  |
| Timing of experiences of interpersonal racism | | 4-level categorical variable |
|  | No experiences of interpersonal racism |  |
|  | Racism over 5 years ago (but not within the past 5 years) |  |
|  | Racism within the past 5 years (but not over 5 years ago) |  |
|  | Both past and recent experiences of racism |  |
| Timing of experiences of institutional racism | | 4-level categorical variable |
|  | No experiences of institutional racism |  |
|  | Racism over 5 years ago (but not within the past 5 years) |  |
|  | Racism within the past 5 years (but not over 5 years ago) |  |
|  | Both past and recent experiences of racism |  |

# **Table S2.** Demographic characteristics of sample (N = 8,897). Weighted percentages are shown.

| **Variable** | | **No CMD N (%)** | **CMD N (%)** | **Total N (%)** |
| --- | --- | --- | --- | --- |
| Sex | |  |  |  |
|  | Female | 2,419 (47.65) | 2,852 (58.09) | 5,271 (52.39) |
|  | Male | 2,048 (52.35) | 1,578 (41.91) | 3,626 (47.61) |
|  | *Missing* | *0 (0.00)* | *0 (0.00)* | *0 (0.00)* |
| Age | |  |  |  |
|  | 18-29 | 1,558 (24.71) | 1,899 (27.27) | 3,457 (27.27) |
|  | 30-44 | 1,942 (43.14) | 1,775 (43.72) | 3,717 (43.72) |
|  | 45-60 | 967 (32.14) | 756 (29.02) | 1,723 (29.02) |
|  | *Missing* | *0 (0.00)* | *0 (0.00)* | *0 (0.00)* |
| Ethnicity | |  |  |  |
|  | Bangladeshi | 163 (3.73) | 228 (4.19) | 391 (3.94) |
|  | Chinese | 335 (4.00) | 312 (3.11) | 647 (3.59) |
|  | Indian | 610 (11.86) | 603 (12.39) | 1,213 (12.10) |
|  | Pakistani | 379 (8.45) | 439 (9.70) | 818 (9.02) |
|  | Any other Asian background | 352 (7.83) | 294 (5.07) | 646 (6.57) |
|  | Black African | 548 (10.46) | 464 (8.43) | 1,012 (9.54) |
|  | Black Caribbean | 242 (3.70) | 280 (4.53) | 522 (4.08) |
|  | Any other Black background | 81 (0.96) | 86 (1.71) | 167 (1.30) |
|  | Mixed White and Asian | 268 (2.27) | 239 (1.89) | 507 (2.10) |
|  | Mixed White and Black African | 62 (1.08) | 92 (0.98) | 154 (1.04) |
|  | Mixed White and Black Caribbean | 139 (2.10) | 201 (3.03) | 340 (2.52) |
|  | Any other Mixed background | 153 (2.08) | 202 (2.49) | 355 (2.27) |
|  | Gypsy/Traveller/Roma | 178 (1.36) | 104 (1.02) | 282 (1.21) |
|  | Any other White background | 544 (32.45) | 501 (31.21) | 1,045 (31.89) |
|  | Arab | 61 (1.63) | 86 (2.64) | 147 (2.09) |
|  | Jewish | 229 (1.42) | 178 (1.11) | 407 (1.28) |
|  | Any other ethnicity | 123 (4.62) | 121 (6.51) | 244 (5.48) |
| Educational attainment | |  |  |  |
|  | Higher education | 2,857 (55.20) | 2,696 (49.58) | 5,553 (52.65) |
|  | A-level or equivalent/vocational qualification | 1,117 (29.79) | 1,250 (32.31) | 2,367 (30.94) |
|  | GCSE or below | 420 (10.50) | 402 (12.92) | 822 (11.60) |
|  | Other | 45 (3.25) | 56 (3.51) | 101 (3.37) |
|  | *Don’t know* | *28 (1.25)* | *26 (1.68)* | *54 (1.45)* |
| Marital status | |  |  |  |
|  | Married or civil partnership | 2,158 (54.50) | 1,562 (42.38) | 3,720 (49.00) |
|  | Never married | 1,937 (36.65) | 2,392 (43.59) | 4,329 (39.80) |
|  | Divorced or separated | 236 (5.07) | 314 (8.76) | 550 (6.75) |
|  | Widowed | 21 (0.94) | 30 (1.29) | 51 (1.10) |
|  | *Prefer not to say* | *115 (2.84)* | *132 (3.98)* | *247 (3.36)* |
| Country of birth | |  |  |  |
|  | UK born | 2,263 (36.06) | 2,597 (45.79) | 4,860 (40.48) |
|  | Foreign born | 2,053 (59.39) | 1,686 (50.61) | 3,739 (55.41) |
|  | *Missing* | *151 (4.55)* | *147 (3.59)* | *298 (4.12)* |

# **Table S3.** Descriptive statistics for experiences during the pandemic. Percentages are weighted.

| **Variable** | | **No CMD N (%)** | **CMD N (%)** | **Total N (%)** |
| --- | --- | --- | --- | --- |
| Previous COVID-19 infection | |  |  |  |
|  | No | 3,891 (87.97) | 3,815 (85.88) | 7,706 (87.02) |
|  | Yes | 560 (11.39) | 590 (13.81) | 1,150 (12.49) |
|  | *Prefer not to say* | *16 (0.64)* | *25 (0.31)* | *41 (0.49)* |
| Changes in household income | |  |  |  |
|  | Higher | 1,172 (25.59) | 1,033 (23.16) | 2,205 (24.49) |
|  | About the same | 1,959 (45.07) | 1,630 (39.20) | 3,589 (42.40) |
|  | Lower | 1,036 (23.57) | 1,407 (32.44) | 2,443 (27.60) |
|  | *Don’t know* | *197 (3.85)* | *236 (2.87)* | *433 (3.41)* |
|  | *Prefer not to say* | *103 (1.92)* | *124 (2.33)* | *227 (2.11)* |
| Worried about future financial security | |  |  |  |
|  | Not at all worried | 1,412 (32.29) | 565 (13.30) | 1,977 (23.67) |
|  | Somewhat worried | 2,383 (50.99) | 2,229 (49.03) | 4,612 (50.10) |
|  | Very/Extremely worried | 542 (12.78) | 1,553 (36.13) | 2,095 (23.38) |
|  | *Prefer not to say* | *130 (3.94)* | *83 (1.54)* | *213 (2.85)* |
| Loneliness & isolation (mean ± SD) | | 2.02 (2.03) | 4.61 (2.24) | 3.29 (2.50) |
| Changes in loneliness & isolation | |  |  |  |
|  | Decreased/Stopped | 847 (20.38) | 824 (20.94) | 1,671 (20.64) |
|  | Stayed the same | 2,373 (55.34) | 1,188 (27.69) | 3,561 (42.78) |
|  | Increased | 1,247 (24.28) | 2,418 (51.37) | 3,665 (36.58) |
|  | *Missing* | *0 (0.00)* | *0 (0.00)* | *0 (0.00)* |
| Belonging to local area | |  |  |  |
|  | Very/Fairly strongly | 3,287 (76.34) | 2,852 (69.35) | 6,139 (73.16) |
|  | Not very/Not at all strongly | 1,079 (21.68) | 1,471 (28.69) | 2,550 (24.86) |
|  | *Don’t know* | *101 (1.99)* | *107 (1.96)* | *208 (1.98)* |
| Changes in belonging | |  |  |  |
|  | Increased | 1,519 (36.27) | 1,422 (38.77) | 2,941 (37.40) |
|  | No change | 2,313 (50.35) | 1,966 (39.20) | 4,279 (45.28) |
|  | Decreased | 509 (10.29) | 917 (18.92) | 1,426 (14.21) |
|  | *Don’t know* | *126 (3.10)* | *125 (3.12)* | *251 (3.11)* |

# **Table S4.** Descriptive statistics for experiences of racism across the life course. Percentages are weighted.

| **Variable** | | **No CMD N (%)** | **CMD N (%)** | **Total N (%)** |
| --- | --- | --- | --- | --- |
| Cumulative experiences of racism | |  |  |  |
|  | No experiences of racism | 1,261 (36.31) | 602 (20.54) | 1,863 (29.15) |
|  | Racism in 1 domain at 1 or 2 time points | 1,013 (21.04) | 713 (15.41) | 1,726 (18.49) |
|  | Racism in 1 domain at 3 or 4 time points | 29 (0.35) | 32 (0.55) | 61 (0.44) |
|  | Racism in 2 domains at 1 or 2 time points | 913 (19.90) | 988 (21.51) | 1,901 (20.63) |
|  | Racism in 2 domains at 3 or 4 time points | 367 (5.42) | 478 (7.38) | 845 (6.31) |
|  | Racism in 3 domains at 1 or 2 time points | 414 (9.24) | 695 (15.68) | 1,109 (12.17) |
|  | Racism in 3 domains at 3 or 4 time points | 470 (7.74) | 922 (18.92) | 1,392 (12.82) |
|  | *Missing* | *0 (0.00)* | *0 (0.00)* | *0 (0.00)* |
| Timing of experiences of racism | |  |  |  |
|  | No experiences of racism | 1,261 (36.31) | 602 (20.54) | 1,863 (29.15) |
|  | Past experiences but not recent | 748 (18.11) | 632 (14.11) | 1,380 (16.29) |
|  | Recent experiences but not past | 1,146 (22.76) | 1,255 (26.79) | 2,401 (24.59) |
|  | Past and recent experiences | 1,312 (22.82) | 1,941 (38.56) | 3,253 (29.96) |
|  | *Missing* | *0 (0.00)* | *0 (0.00)* | *0 (0.00)* |
| Timing of experiences of hate crimes | |  |  |  |
|  | No experiences | 3,334 (76.81) | 2,566 (60.21) | 5,900 (69.28) |
|  | Past experiences but not recent | 465 (10.09) | 639 (13.50) | 1,104 (11.64) |
|  | Recent experiences but not past | 574 (10.96) | 1,054 (23.21) | 1,628 (16.52) |
|  | Past and recent experiences | 28 (0.61) | 96 (1.46) | 124 (1.00) |
|  | *Missing* | *66 (1.53)* | *75 (1.61)* | *141 (1.57)* |
| Timing of experiences of interpersonal racism | |  |  |  |
|  | No experiences | 1,452 (41.25) | 725 (23.84) | 2,177 (33.34) |
|  | Past experiences but not recent | 768 (18.12) | 740 (16.70) | 1,508 (17.48) |
|  | Recent experiences but not past | 1,945 (36.06) | 2,487 (51.49) | 4,423 (43.07) |
|  | Past and recent experiences | 281 (4.28) | 472 (18.05) | 753 (5.76) |
|  | *Missing* | *21 (0.29)* | *15 (0.43)* | *36 (0.36)* |
| Timing of experiences of institutional racism | |  |  |  |
|  | No experiences | 2,232 (56.54) | 1,329 (35.47) | 3,561 (46.97) |
|  | Past experiences but not recent | 681 (15.10) | 718 (15.83) | 1,399 (15.43) |
|  | Recent experiences but not past | 1,355 (25.35) | 2,026 (43.23) | 3,381 (33.47) |
|  | Past and recent experiences | 155 (2.40) | 302 (4.45) | 457 (3.33) |
|  | *Missing* | *44 (0.61)* | *55 (1.03)* | *99 (0.80)* |

# **Table S5.** Tetrachoric correlation matrix for each domain of racism (yes at any time point vs no at all time points): hate crimes, interpersonal racism, institutional racism (N = 8,796).

|  | **Hate crime** | **Interpersonal** | **Institutional** |
| --- | --- | --- | --- |
| **Hate crime** | 1.00 |  |  |
| **Interpersonal** | 0.78*** | 1.00 |  |
| **Institutional** | 0.68*** | 0.78*** | 1.00 |

*p<0.05, **p<0.01, ***p<0.001

# **Table S6.** Independent associations between each of the exposure variables and the health impacts of the pandemic. Weighted and adjusted multinomial odds ratios (MOR) are presented, with 95% Confidence Intervals (CIs) (N = 9,019).

|  | | **Previous infection** |
| --- | --- | --- |
| **Variable** | | **“Yes”** |
| Cumulative experiences of racism | |  |
|  | No experiences of racism | 1.00 |
|  | Racism in 1 domain at 1 or 2 time points | 0.89 (0.63 to 1.27) |
|  | Racism in 1 domain at 3 or 4 time points | 0.73 (0.25 to 2.18) |
|  | Racism in 2 domains at 1 or 2 time points | 1.25 (0.90 to 1.72) |
|  | Racism in 2 domains at 3 or 4 time points | 0.78 (0.52 to 1.18) |
|  | Racism in 3 domains at 1 or 2 time points | 1.88 (1.29 to 2.73)** |
|  | Racism in 3 domains at 3 or 4 time points | 1.45 (1.01 to 2.04)* |
| Timing of experiences of racism | |  |
|  | No experiences of racism | 1.00 |
|  | Past experiences but not recent | 1.01 (0.70 to 1.45) |
|  | Recent experiences but not past | 1.35 (0.99 to 1.85) |
|  | Past and recent experiences | 1.20 (0.90 to 1.62) |
| Timing of experiences of hate crimes | |  |
|  | No experiences of racism | 1.00 |
|  | Past experiences but not recent | 1.37 (0.97 to 1.92) |
|  | Recent experiences but not past | 1.77 (1.32 to 2.35)*** |
|  | Past and recent experiences | 0.95 (0.41 to 2.19) |
| Timing of experiences of interpersonal racism | |  |
|  | No experiences of racism | 1.00 |
|  | Past experiences but not recent | 1.04 (0.75 to 1.46) |
|  | Recent experiences but not past | 1.42 (1.09 to 1.85)** |
|  | Past and recent experiences | 0.77 (0.50 to 1.20) |
| Timing of experiences of institutional racism | |  |
|  | No experiences of racism | 1.00 |
|  | Past experiences but not recent | 1.05 (0.75 to 1.47) |
|  | Recent experiences but not past | 1.40 (1.10 to 1.79)** |
|  | Past and recent experiences | 1.18 (0.73 to 1.93) |

Reference group = “No”. “Prefer not to say” and “Don’t know” responses are not shown. Adjusted analyses control for age, age squared, sex, educational attainment, marital status, country of birth, and month when survey was completed.

# **Table S7.** Independent associations between each of the exposure variables and the economic impacts of the pandemic. Weighted and adjusted multinomial odds ratios (MOR) are presented, with 95% Confidence Intervals (CIs) (N = 9,019).

|  | | **Changes in income** | | **Worried about future financial situation** | |
| --- | --- | --- | --- | --- | --- |
| **Variable** | | **“Higher”** | **“Lower”** | **“Somewhat”** | **“Very/Extremely”** |
| Cumulative experiences of racism | |  |  |  |  |
|  | No experiences of racism | 1.00 | 1.00 | 1.00 | 1.00 |
|  | Racism in 1 domain at 1 or 2 time points | 1.05 (0.78 to 1.43) | 1.28 (0.95 to 1.72) | 1.34 (1.03 to 1.74)* | 1.46 (0.98 to 2.16) |
|  | Racism in 1 domain at 3 or 4 time points | 1.31 (0.47 to 3.64) | 2.94 (0.92 to 9.33) | 5.22 (1.62 to 16.78)** | / |
|  | Racism in 2 domains at 1 or 2 time points | 1.13 (0.83 to 1.54) | 1.44 (1.08 to 1.91)* | 1.60 (1.18 to 2.16)** | 2.15 (1.47 to 3.14)*** |
|  | Racism in 2 domains at 3 or 4 time points | 0.79 (0.53 to 1.18) | 1.23 (0.84 to 1.79) | 1.67 (1.16 to 2.40)** | 2.21 (1.41 to 3.46)** |
|  | Racism in 3 domains at 1 or 2 time points | 2.43 (1.74 to 3.39)*** | 2.33 (1.66 to 3.26)*** | 1.56 (1.10 to 2.21)* | 4.05 (2.68 to 6.12)*** |
|  | Racism in 3 domains at 3 or 4 time points | 1.70 (1.26 to 2.30)** | 1.58 (1.16 to 2.16)** | 2.37 (1.73 to 3.26)*** | 4.16 (2.82 to 6.13)*** |
| Timing of experiences of racism | |  |  |  |  |
|  | No experiences of racism | 1.00 | 1.00 | 1.00 | 1.00 |
|  | Past experiences but not recent | 1.15 (0.83 to 1.58) | 1.26 (0.93 to 1.72) | 1.25 (0.93 to 1.69) | 1.43 (0.97 to 2.12) |
|  | Recent experiences but not past | 1.48 (1.11 to 1.97)** | 1.71 (1.30 to 2.26)*** | 1.55 (1.19 to 2.01)** | 2.55 (1.80 to 3.61)*** |
|  | Past and recent experiences | 1.23 (0.95 to 1.59) | 1.47 (1.13 to 1.90)** | 1.99 (1.54 to 2.58)*** | 3.15 (2.26 to 4.38)*** |
| Timing of experiences of hate crimes | |  |  |  |  |
|  | No experiences of racism | 1.00 | 1.00 | 1.00 | 1.00 |
|  | Past experiences but not recent | 1.34 (1.00 to 1.80)* | 1.18 (0.87 to 1.59) | 1.22 (0.86 to 1.73) | 1.42 (0.95 to 2.14) |
|  | Recent experiences but not past | 2.17 (1.68 to 2.80)*** | 1.83 (1.41 to 2.38)*** | 1.54 (1.17 to 2.04)** | 3.33 (2.42 to 4.58)*** |
|  | Past and recent experiences | 1.22 (0.61 to 2.45) | 1.60 (0.82 to 3.11) | 1.69 (0.65 to 4.37) | 4.89 (1.88 to 12.72)** |
| Timing of experiences of interpersonal racism | |  |  |  |  |
|  | No experiences of racism | 1.00 | 1.00 | 1.00 | 1.00 |
|  | Past experiences but not recent | 1.20 (0.88 to 1.63) | 1.27 (0.95 to 1.70) | 1.21 (0.90 to 1.61) | 1.46 (1.01 to 2.13)* |
|  | Recent experiences but not past | 1.37 (1.08 to 1.74)* | 1.40 (1.11 to 1.76)** | 1.56 (1.24 to 1.96)*** | 2.41 (1.79 to 3.25)*** |
|  | Past and recent experiences | 1.23 (0.83 to 1.82) | 1.65 (1.13 to 2.41)* | 2.06 (1.40 to 3.02)*** | 3.13 (1.99 to 4.92)*** |
| Timing of experiences of institutional racism | |  |  |  |  |
|  | No experiences of racism | 1.00 | 1.00 | 1.00 | 1.00 |
|  | Past experiences but not recent | 1.03 (0.78 to 1.36) | 1.05 (0.80 to 1.38) | 1.43 (1.10 to 1.86)** | 1.93 (1.38 to 2.68)*** |
|  | Recent experiences but not past | 1.61 (1.29 to 2.00)*** | 1.72 (1.39 to 2.14)*** | 1.86 (1.50 to 2.32)*** | 3.25 (2.54 to 4.43)*** |
|  | Past and recent experiences | 1.26 (0.78 to 2.02) | 1.88 (1.22 to 2.88)** | 1.40 (0.90 to 2.18) | 2.42 (1.48 to 3.97)*** |

Reference groups: changes in income = “Stayed the same”, worried about future financial situation = “not at all worried”. “Prefer not to say” and “Don’t know” not shown.

#

# **Table S8.** Independent associations between each of the exposure variables and loneliness. Beta values (β) and adjusted multinomial odds ratios (MOR) are presented, with 95% Confidence Intervals (CIs) (N = 9,019). Analyses are weighted.

|  | | **Loneliness** | **Change in loneliness** | |
| --- | --- | --- | --- | --- |
| **Variable** | | **β** | **“Stayed the same”** | **“Increased”** |
| Cumulative experiences of racism | |  |  |  |
|  | No experiences of racism | 0.00 | 1.00 | 1.00 |
|  | Racism in 1 domain at 1 or 2 time points | 0.52 (0.26 to 0.77)*** | 0.83 (0.58 to 1.19) | 1.11 (0.77 to 1.60) |
|  | Racism in 1 domain at 3 or 4 time points | 1.18 (-0.34 to 2.68) | 0.99 (0.19 to 5.09) | 2.37 (0.55 to 10.28) |
|  | Racism in 2 domains at 1 or 2 time points | 0.95 (0.69 to 1.20)*** | 0.80 (0.57 to 1.14) | 1.57 (1.10 to 2.23)* |
|  | Racism in 2 domains at 3 or 4 time points | 1.20 (0.84 to 1.56)*** | 1.48 (0.94 to 2.34) | 3.12 (1.98 to 4.90)*** |
|  | Racism in 3 domains at 1 or 2 time points | 1.58 (1.30 to 1.86)*** | 0.28 (0.20 to 0.40)*** | 0.75 (0.52 to 1.07) |
|  | Racism in 3 domains at 3 or 4 time points | 1.73 (1.47 to 1.99)*** | 0.32 (0.23 to 0.45)*** | 0.82 (0.58 to 1.14) |
| Timing of experiences of racism | |  |  |  |
|  | No experiences of racism | 0.00 | 1.00 | 1.00 |
|  | Past experiences but not recent | 0.73 (0.47 to 1.00)*** | 0.86 (0.59 to 1.25) | 1.25 (0.85 to 1.84) |
|  | Recent experiences but not past | 0.98 (0.75 to 1.21)*** | 0.53 (0.38 to 0.73)*** | 1.01 (0.73 to 1.39) |
|  | Past and recent experiences | 1.36 (1.13 to 1.59)*** | 0.54 (0.40 to 0.73)*** | 1.22 (0.91 to 1.65) |
| Timing of experiences of hate crimes | |  |  |  |
|  | No experiences of racism | 0.00 | 1.00 | 1.00 |
|  | Past experiences but not recent | 0.91 (0.64 to 1.17)*** | 0.50 (0.35 to 0.72)*** | 0.68 (0.49 to 0.96)* |
|  | Recent experiences but not past | 1.13 (0.90 to 1.35)*** | 0.30 (0.23 to 0.39)*** | 0.56 (0.43 to 0.72)*** |
|  | Past and recent experiences | 1.45 (0.81 to 2.09)*** | 0.35 (0.17 to 0.75)** | 0.81 (0.42 to 1.55) |
| Timing of experiences of interpersonal racism | |  |  |  |
|  | No experiences of racism | 0.00 | 1.00 | 1.00 |
|  | Past experiences but not recent | 0.64 (0.38 to 0.91)*** | 1.01 (0.71 to 1.44) | 1.60 (1.12 to 2.28)* |
|  | Recent experiences but not past | 1.06 (0.86 to 1.27)*** | 0.60 (0.46 to 0.78)*** | 1.22 (0.93 to 1.59) |
|  | Past and recent experiences | 1.25 (0.94 to 1.56)*** | 1.08 (0.68 to 1.72) | 2.23 (1.42 to 3.50)** |
| Timing of experiences of institutional racism | |  |  |  |
|  | No experiences of racism | 0.00 | 1.00 | 1.00 |
|  | Past experiences but not recent | 0.85 (0.61 to 1.09)*** | 0.69 (0.51 to 0.95)* | 1.14 (0.83 to 1.57) |
|  | Recent experiences but not past | 1.15 (0.96 to 1.34)*** | 0.42 (0.33 to 0.54)*** | 0.87 (0.68 to 1.12) |
|  | Past and recent experiences | 1.45 (1.09 to 1.81)*** | 0.49 (0.30 to 0.79)** | 1.10 (0.68 to 1.78) |

Reference groups: changes in loneliness = “stopped”. “Prefer not to say” and “Don’t know” not shown.

# **Table S9.** Independent associations between each of the exposure variables and belonging. Weighted and adjusted multinomial odds ratios (MOR) are presented, with 95% Confidence Intervals (CIs) (N = 8,888).

|  | | **Belonging** | **Change in belonging** |  |
| --- | --- | --- | --- | --- |
| **Variable** | | **“Not strongly”** | **“Increased”** | **“Decreased”** |
| Cumulative experiences of racism | |  |  |  |
|  | No experiences of racism | 1.00 | 1.00 | 1.00 |
|  | Racism in 1 domain at 1 or 2 time points | 1.16 (0.89 to 1.51) | 1.04 (0.81 to 1.34) | 1.59 (1.06 to 2.38)* |
|  | Racism in 1 domain at 3 or 4 time points | 1.77 (0.52 to 5.96) | 2.40 (0.80 to 7.23) | 2.76 (0.42 to 18.02) |
|  | Racism in 2 domains at 1 or 2 time points | 1.21 (0.92 to 1.60) | 1.00 (0.77 to 1.29) | 1.73 (1.19 to 2.50)** |
|  | Racism in 2 domains at 3 or 4 time points | 1.64 (1.17 to 2.29)** | 0.98 (0.69 to 1.40) | 1.53 (1.00 to 2.34) |
|  | Racism in 3 domains at 1 or 2 time points | 1.02 (0.75 to 1.37) | 2.03 (1.53 to 2.71)*** | 2.52 (1.66 to 3.80)*** |
|  | Racism in 3 domains at 3 or 4 time points | 1.61 (1.21 to 2.15)** | 1.57 (1.20 to 2.07)** | 2.44 (1.66 to 3.59)*** |
| Timing of experiences of racism | |  |  |  |
|  | No experiences of racism | 1.00 | 1.00 | 1.00 |
|  | Past experiences but not recent | 1.05 (0.77 to 1.45) | 0.99 (0.75 to 1.30) | 1.06 (0.72 to 1.57) |
|  | Recent experiences but not past | 1.22 (0.95 to 1.56) | 1.26 (1.00 to 1.60) | 2.18 (1.50 to 3.17)*** |
|  | Past and recent experiences | 1.43 (1.13 to 1.79)** | 1.34 (1.07 to 1.68)* | 2.06 (1.48 to 2.85)*** |
| Timing of experiences of hate crimes | |  |  |  |
|  | No experiences of racism | 1.00 | 1.00 | 1.00 |
|  | Past experiences but not recent | 1.26 (0.92 to 1.72) | 1.04 (0.80 to 1.36) | 1.11 (0.79 to 1.56) |
|  | Recent experiences but not past | 1.12 (0.89 to 1.40) | 2.18 (1.73 to 2.74)*** | 2.43 (1.79 to 3.28)*** |
|  | Past and recent experiences | 1.21 (0.66 to 2.23) | 1.51 (0.79 to 2.89) | 2.31 (1.11 to 4.80)* |
| Timing of experiences of interpersonal racism | |  |  |  |
|  | No experiences of racism | 1.00 | 1.00 | 1.00 |
|  | Past experiences but not recent | 1.13 (0.84 to 1.52) | 0.98 (0.76 to 1.27) | 1.03 (0.70 to 1.51) |
|  | Recent experiences but not past | 1.19 (0.97 to 1.47) | 1.30 (1.06 to 1.58)* | 1.83 (1.34 to 2.49)*** |
|  | Past and recent experiences | 2.07 (1.47 to 2.91)*** | 0.94 (0.66 to 1.34) | 1.72 (1.12 to 2.62)** |
| Timing of experiences of institutional racism | |  |  |  |
|  | No experiences of racism | 1.00 | 1.00 | 1.00 |
|  | Past experiences but not recent | 1.01 (0.78 to 1.31) | 1.05 (0.82 to 1.39) | 1.13 (0.83 to 1.54) |
|  | Recent experiences but not past | 1.21 (1.00 to 1.47) | 1.50 (1.24 to 1.81)*** | 1.90 (1.44 to 2.51)*** |
|  | Past and recent experiences | 1.93 (1.31 to 2.84)** | 1.24 (0.82 to 1.88) | 1.96 (1.25 to 3.08)** |

Reference groups: belonging = “Strongly”, change in belonging = “not changed”. “Prefer not to say” and “Don’t know” not shown.

# **Table S10.** Independent associations between each of the potential pathway variables and CMD during the pandemic. Unadjusted and adjusted odds ratios (OR) are presented, with 95% Confidence Intervals (CIs). Analyses are weighted to account for selection bias and coverage bias.

| **Variable** | | **OR (95% CI)**  **N = 8897** | **aOR (95% CI)**  **N = 8888** |
| --- | --- | --- | --- |
| Previous COVID-19 infection | |  |  |
|  | No | 1.00 | 1.00 |
|  | Yes | 1.24 (1.00 to 1.54)* | 1.21 (0.97 to 1.51) |
| Changes in household income | |  |  |
|  | Higher | 1.04 (0.86 to 1.26) | 0.99 (0.82 to 1.21) |
|  | About the same | 1.00 | 1.00 |
|  | Lower | 1.58 (1.31 to 1.92)*** | 1.56 (1.28 to 1.89)*** |
| Worried about future financial security | |  |  |
|  | Not at all worried | 1.00 | 1.00 |
|  | Somewhat worried | 2.34 (1.90 to 2.88)*** | 2.41 (1.96 to 2.96)*** |
|  | Very/Extremely worried | 6.87 (5.29 to 8.91)*** | 7.21 (5.57 to 9.35)*** |
| Loneliness & isolation (mean ± SD) | | 1.68 (1.61 to 1.75)*** | 1.67 (1.60 to 1.75)*** |
| Changes in loneliness & isolation | |  |  |
|  | Stopped/Decreased | 1.00 | 1.00 |
|  | Stayed the same | 0.49 (0.39 to 0.61)*** | 0.54 (0.43 to 0.68)*** |
|  | Increased | 2.06 (1.66 to 2.55)*** | 2.27 (1.82 to 2.83)*** |
| Belonging to local area | |  |  |
|  | Very/fairly strongly | 1.00 | 1.00 |
|  | Not very/at all strongly | 1.46 (1.22 to 1.74)*** | 1.51 (1.26 to 1.80)*** |
| Changes in belonging | |  |  |
|  | Increased | 1.37 (1.16 to 1.62)*** | 1.39 (1.17 to 1.65)*** |
|  | No change | 1.00 | 1.00 |
|  | Decreased | 2.36 (1.85 to 3.02)*** | 2.22 (1.73 to 2.85)*** |

Adjusted analyses control for age, age squared, sex, educational attainment, marital status, country of birth, and month when survey was completed. “Prefer not to say” and “Don’t know” not shown.

# **Table S11.** Complete case path analysis estimating the relationship between timing of experiences of racism and CMD, through potential pathways (N = 8,421). Analyses are weighted to account for selection bias and coverage bias.

|  | | **CMD** | | **Past experiences** | | **Recent experiences** | | **Past & Recent** | |
| --- | --- | --- | --- | --- | --- | --- | --- | --- | --- |
|  | | **aOR** | **95% CI** | **aOR** | **95% CI** | **aOR** | **95% CI** | **aOR** | **95% CI** |
| *Path α* | |  |  |  |  |  |  |  |  |
|  | Racism 🡪 previous infection *α1* |  |  | 1.00 |  | 1.00 |  | 1.00 |  |
|  | *Yes* |  |  | 1.04 | 0.72 to 1.50 | 1.44* | 1.04 to 1.98 | 1.28 | 0.94 to 1.73 |
|  | Racism 🡪 change in income *α2* |  |  | 1.00 |  | 1.00 |  | 1.00 |  |
|  | *About the same* |  |  | 0.89 | 0.64 to 1.23 | 0.66** | 0.49 to 0.88 | 0.81 | 0.62 to 1.06 |
|  | *Lower* |  |  | 1.11 | 0.77 to 1.60 | 1.10 | 0.80 to 1.51 | 1.23 | 0.91 to 1.65 |
|  | Racism 🡪 financial concerns *α3* |  |  | 1.00 |  | 1.00 |  | 1.00 |  |
|  | *Somewhat concerned* |  |  | 1.28 | 0.94 to 1.75 | 1.53** | 1.17 to 2.00 | 1.96*** | 1.50 to 2.56 |
|  | *Very/extremely concerned* |  |  | 1.55* | 1.04 to 2.32 | 2.54*** | 1.77 to 3.65 | 3.14*** | 2.22 to 4.43 |
|  | Racism 🡪 loneliness *α4 (βeta coefficient)* |  |  | 0.90*** | 0.63 to 1.18 | 1.13*** | 0.91 to 1.36 | 1.57*** | 0.87 to 2.28 |
|  | Racism 🡪 change in loneliness *α5* |  |  | 1.00 |  | 1.00 |  | 1.00 |  |
|  | *Stayed the same* |  |  | 0.50*** | 0.34 to 0.72 | 0.32*** | 0.24 to 0.42 | 0.32** | 0.14 to 0.74 |
|  | *Increased* |  |  | 0.65* | 0.46 to 0.92 | 0.57*** | 0.45 to 0.76 | 0.82 | 0.40 to 1.66 |
|  | Racism 🡪 Sense of belonging *α6* |  |  | 1.00 |  | 1.00 |  | 1.00 |  |
|  | *Not very/not at all* |  |  | 1.05 | 0.95 to 1.17 | 1.19 | 0.92 to 1.54 | 1.42** | 1.12 to 1.81 |
|  | Racism 🡪 change in belonging *α7* |  |  | 1.00 |  | 1.00 |  | 1.00 |  |
|  | *Increased* |  |  | 0.97 | 0.73 to 1.29 | 1.27 | 1.00 to 1.62 | 1.36** | 1.08 to 1.71 |
|  | *Decreased* |  |  | 1.07 | 0.71 to 1.61 | 2.16*** | 1.47 to 3.18 | 2.12*** | 1.49 to 3.01 |
| *Path β* | |  |  |  |  |  |  |  |  |
|  | Previous infection 🡪 CMD *β1* | 1.00 |  |  |  |  |  |  |  |
|  | *Yes* | 1.30 | 0.96 to 1.75 |  |  |  |  |  |  |
|  | Change in income 🡪 CMD *β2* | 1.00 |  |  |  |  |  |  |  |
|  | *About the same* | 1.07 | 0.83 to 1.37 |  |  |  |  |  |  |
|  | *Lower* | 1.02 | 0.77 to 1.35 |  |  |  |  |  |  |
|  | Financial concerns 🡪 CMD *β3* | 1.00 |  |  |  |  |  |  |  |
|  | *Somewhat concerned* | 1.87*** | 1.44 to 2.41 |  |  |  |  |  |  |
|  | *Very/extremely concerned* | 4.43*** | 3.13 to 6.26 |  |  |  |  |  |  |
|  | Loneliness 🡪 CMD *β4* | 1.52*** | 1.44 to 1.60 |  |  |  |  |  |  |
|  | Change in loneliness 🡪 CMD *β5* | 1.00 |  |  |  |  |  |  |  |
|  | *Stayed the same* | 0.85 | 0.64 to 1.13 |  |  |  |  |  |  |
|  | *Increased* | 1.59** | 1.19 to 2.12 |  |  |  |  |  |  |
|  | Sense of belonging 🡪 CMD *β6* | 1.00 |  |  |  |  |  |  |  |
|  | *Not very/not at all* | 1.56*** | 1.23 to 1.98 |  |  |  |  |  |  |
|  | Change in belonging 🡪 CMD *β7* | 1.00 |  |  |  |  |  |  |  |
|  | *Increased* | 1.13** | 1.18 to 1.82 |  |  |  |  |  |  |
|  | *Decreased* | 1.46* | 1.05 to 2.03 |  |  |  |  |  |  |
| *Path α1*β1* | |  |  |  |  |  |  |  |  |
|  | Racism 🡪 CMD (indirect through previous infection) |  |  | 1.00 |  | 1.00 |  | 1.00 |  |
|  | *Yes* |  |  | 1.14 | 0.77 to 1.51 | 1.62* | 1.07 to 2.17 | 1.89* | 1.31 to 2.46 |
| *Path α2*β2* | |  |  |  |  |  |  |  |  |
|  | Racism 🡪 CMD (indirect through change in income) |  |  | 1.00 |  | 1.00 |  | 1.00 |  |
|  | *About the same* |  |  | 1.12 | 0.77 to 1.47 | 1.43 | 0.97 to 1.89 | 1.74* | 1.25 to 2.24 |
|  | *Lower* |  |  | 1.13 | 0.78 to 1.49 | 1.47* | 1.03 to 1.92 | 1.77* | 1.26 to 2.28 |
| *Path α3*β3* | |  |  |  |  |  |  |  |  |
|  | Racism 🡪 CMD (indirect through financial concerns) |  |  | 1.00 |  | 1.00 |  | 1.00 |  |
|  | *Somewhat concerned* |  |  | 1.32 | 0.83 to 1.82 | 1.91* | 1.24 to 2.60 | 2.69* | 1.69 to 3.68 |
|  | *Very/extremely concerned* |  |  | 2.17 | 0.68 to 2.67 | 5.89* | 1.87 to 9.91 | 9.67* | 2.86 to 16.49 |
| *Path α4*β4* | |  |  |  |  |  |  |  |  |
|  | Racism 🡪 CMD (indirect through loneliness) |  |  | 1.51 | 1.00 to 2.02 | 2.18* | 1.50 to 2.87 | 3.03* | 2.14 to 3.92 |
| *Path α5*β5* | |  |  |  |  |  |  |  |  |
|  | Racism 🡪 CMD (indirect through change in loneliness) |  |  | 1.00 |  | 1.00 |  | 1.00 |  |
|  | *Stayed the same* |  |  | 1.16 | 0.79 to 1.52 | 1.61* | 1.06 to 2.16 | 1.93* | 1.30 to 2.57 |
|  | *Increased* |  |  | 1.23 | 0.77 to 1.70 | 1.50 | 0.97 to 2.02 | 1.95* | 1.32 to 2.57 |
| *Path α6*β6* | |  |  |  |  |  |  |  |  |
|  | Racism 🡪 CMD (indirect through belonging) |  |  | 1.00 |  | 1.00 |  | 1.00 |  |
|  | *Not very/not at all* |  |  | 1.15 | 0.76 to 1.55 | 1.59* | 1.08 to 2.10 | 2.07* | 1.41 to 2.73 |
| *Path α7*β7* | |  |  |  |  |  |  |  |  |
|  | Racism 🡪 CMD (indirect through change in belonging) |  |  | 1.00 |  | 1.00 |  | 1.00 |  |
|  | *Increased* |  |  | 1.12 | 0.75 to 1.49 | 1.61* | 1.09 to 2.14 | 1.98* | 1.38 to 2.60 |
|  | *Decreased* |  |  | 1.16 | 0.75 to 1.57 | 1.97* | 1.12 to 2.81 | 2.35* | 1.42 to 3.27 |
| *Path τ* | |  |  |  |  |  |  |  |  |
|  | Total effects |  |  | 1.55** | 1.19 to 2.01 | 2.10*** | 1.65 to 2.67 | 2.92*** | 2.32 to 3.67 |
| *Path τ’* | |  |  |  |  |  |  |  |  |
|  | Direct effects |  |  | 1.13 | 0.83 to 1.54 | 1.47* | 1.09 to 1.98 | 1.77*** | 1.34 to 2.34 |

N for each response: CMD = 7,350; timing of experiences of racism = 8,421; previous infection = 8,394; change in household income = 7,860; financial concerns = 8,265; loneliness and isolation = 8,421; change in loneliness and isolation = 8,421; belonging = 8,246; change in belonging = 8,203.

Reference groups: previous infection = no; change in household income = higher; financial concerns = not at all worried; changes in loneliness = decreased/stopped; belonging = very/fairly strongly; change in belonging = no change.

For indirect effects, statistical significance was determined as confidence intervals without the value of 1.

# **Table S12.** Path analysis estimating the relationship between experiences of hate crimes and CMD, through potential pathways (N = 8,421). Analyses are weighted to account for selection bias and coverage bias.

|  | | **CMD** | | **Past experiences** | | **Recent experiences** | | **Past & Recent** | |
| --- | --- | --- | --- | --- | --- | --- | --- | --- | --- |
|  | | **aOR** | **95% CI** | **aOR** | **95% CI** | **aOR** | **95% CI** | **aOR** | **95% CI** |
| *Path α* | |  |  |  |  |  |  |  |  |
|  | Hate crime 🡪 previous infection *α1* |  |  | 1.00 |  | 1.00 |  | 1.00 |  |
|  | *Yes* |  |  | 1.36 | 0.95 to 1.93 | 1.79*** | 1.34 to 2.41 | 0.95 | 0.39 to 2.30 |
|  | Hate crime 🡪 change in income *α2* |  |  | 1.00 |  | 1.00 |  | 1.00 |  |
|  | *About the same* |  |  | 0.80 | 0.59 to 1.08 | 0.47*** | 0.36 to 0.61 | 0.90 | 0.43 to 1.90 |
|  | *Lower* |  |  | 0.94 | 0.67 to 1.30 | 0.81 | 0.61 to 1.07 | 1.26 | 0.56 to 2.82 |
|  | Hate crime 🡪 financial concerns *α3* |  |  | 1.00 |  | 1.00 |  | 1.00 |  |
|  | *Somewhat concerned* |  |  | 1.24 | 0.86 to 1.79 | 1.50** | 1.12 to 1.99 | 1.73 | 0.64 to 4.69 |
|  | *Very/extremely concerned* |  |  | 1.53 | 1.00 to 2.36 | 3.29*** | 2.37 to 4.58 | 4.48** | 1.62 to 12.34 |
|  | Hate crime 🡪 loneliness *α4 (βeta coefficient)* |  |  | 0.69*** | 0.42 to 0.97 | 0.94*** | 0.70 to 1.19 | 1.29*** | 1.06 to 1.52 |
|  | Hate crime 🡪 change in loneliness *α5* |  |  | 1.00 |  | 1.00 |  | 1.00 |  |
|  | *Stayed the same* |  |  | 0.87 | 0.59 to 1.28 | 0.58** | 0.41 to 0.80 | 0.57** | 0.42 to 0.79 |
|  | *Increased* |  |  | 1.21 | 0.81 to 1.80 | 1.04 | 0.74 to 1.45 | 1.23 | 0.89 to 1.69 |
|  | Hate crime 🡪 Sense of belonging *α6* |  |  | 1.00 |  | 1.00 |  | 1.00 |  |
|  | *Not very/not at all* |  |  | 1.26 | 0.91 to 1.75 | 1.08 | 0.85 to 1.36 | 1.04 | 0.55 to 1.97 |
|  | Hate crime 🡪 change in belonging *α7* |  |  | 1.00 |  | 1.00 |  | 1.00 |  |
|  | *Increased* |  |  | 1.07 | 0.81 to 1.41 | 2.30*** | 1.82 to 2.92 | 1.80 | 0.93 to 3.49 |
|  | *Decreased* |  |  | 1.15 | 0.80 to 1.64 | 2.77*** | 2.03 to 3.79 | 2.44* | 1.15 to 5.18 |
| *Path β* | |  |  |  |  |  |  |  |  |
|  | Previous infection 🡪 CMD *β1* | 1.00 |  |  |  |  |  |  |  |
|  | *Yes* | 1.21 | 0.89 to 1.64 |  |  |  |  |  |  |
|  | Change in income 🡪 CMD *β2* | 1.00 |  |  |  |  |  |  |  |
|  | *About the same* | 1.07 | 0.84 to 1.38 |  |  |  |  |  |  |
|  | *Lower* | 1.07 | 0.81 to 1.42 |  |  |  |  |  |  |
|  | Financial concerns 🡪 CMD *β3* | 1.00 |  |  |  |  |  |  |  |
|  | *Somewhat concerned* | 1.83*** | 1.42 to 2.37 |  |  |  |  |  |  |
|  | *Very/extremely concerned* | 4.12*** | 2.90 to 5.84 |  |  |  |  |  |  |
|  | Loneliness 🡪 CMD *β4* | 1.55*** | 1.47 to 1.63 |  |  |  |  |  |  |
|  | Change in loneliness 🡪 CMD *β5* | 1.00 |  |  |  |  |  |  |  |
|  | *Stayed the same* | 0.91 | 0.69 to 1.21 |  |  |  |  |  |  |
|  | *Increased* | 1.76*** | 1.32 to 2.33 |  |  |  |  |  |  |
|  | Sense of belonging 🡪 CMD *β6* | 1.00 |  |  |  |  |  |  |  |
|  | *Not very/not at all* | 1.46*** | 1.32 to 2.33 |  |  |  |  |  |  |
|  | Change in belonging 🡪 CMD *β7* | 1.00 |  |  |  |  |  |  |  |
|  | *Increased* | 1.44** | 1.16 to 1.80 |  |  |  |  |  |  |
|  | *Decreased* | 1.45* | 1.03 to 2.04 |  |  |  |  |  |  |
| *Path α1*β1* | |  |  |  |  |  |  |  |  |
|  | Hate crime 🡪 CMD (indirect through previous infection) |  |  | 1.00 |  | 1.00 |  | 1.00 |  |
|  | *Yes* |  |  | 1.28 | 0.87 to 1.69 | 1.92* | 1.27 to 2.57 | 3.51* | 1.04 to 5.97 |
| *Path α2*β2* | |  |  |  |  |  |  |  |  |
|  | Hate crime 🡪 CMD (indirect through change in income) |  |  | 1.00 |  | 1.00 |  | 1.00 |  |
|  | *About the same* |  |  | 1.19 | 0.82 to 1.56 | 1.63* | 1.09 to 2.17 | 3.52* | 1.26 to 5.77 |
|  | *Lower* |  |  | 1.21 | 0.84 to 1.57 | 1.70* | 1.21 to 2.18 | 3.60* | 1.26 to 5.93 |
| *Path α3*β3* | |  |  |  |  |  |  |  |  |
|  | Hate crime 🡪 CMD (indirect through financial concerns) |  |  | 1.00 |  | 1.00 |  | 1.00 |  |
|  | *Somewhat concerned* |  |  | 1.38 | 0.83 to 1.94 | 2.20* | 1.42 to 2.97 | 4.93 | 0.07 to 0.79 |
|  | *Very/extremely concerned* |  |  | 2.22 | 0.60 to 3.84 | 9.30* | 3.21 to 15.38 | 29.53 | -22.41 to 81.47 |
| *Path α4*β4* | |  |  |  |  |  |  |  |  |
|  | Hate crime 🡪 CMD (indirect through loneliness) |  |  | 1.80* | 1.23 to 2.37 | 2.83* | 1.97 to 3.68 | 7.05* | 1.44 to 12.66 |
| *Path α5*β5* | |  |  |  |  |  |  |  |  |
|  | Hate crime 🡪 CMD (indirect through change in loneliness) |  |  | 1.00 |  | 1.00 |  | 1.00 |  |
|  | *Stayed the same* |  |  | 1.29 | 0.81 to 1.78 | 1.92* | 1.10 to 2.74 | 3.95 | 0.89 to 7.00 |
|  | *Increased* |  |  | 0.95 | 0.55 to 1.35 | 1.27 | 0.81 to 1.74 | 3.16* | 1.11 to 5.22 |
| *Path α6*β6* | |  |  |  |  |  |  |  |  |
|  | Hate crime 🡪 CMD (indirect through belonging) |  |  | 1.00 |  | 1.00 |  | 1.00 |  |
|  | *Not very/not at all* |  |  | 1.32 | 0.86 to 1.78 | 1.77* | 1.24 to 2.30 | 3.60* | 1.22 to 5.98 |
| *Path α7*β7* | |  |  |  |  |  |  |  |  |
|  | Hate crime 🡪 CMD (indirect through change in belonging) |  |  | 1.00 |  | 1.00 |  | 1.00 |  |
|  | *Increased* |  |  | 1.24 | 0.84 to 1.65 | 2.34* | 1.56 to 3.12 | 4.40* | 1.48 to 7.31 |
|  | *Decreased* |  |  | 1.27 | 0.84 to 1.71 | 2.52* | 1.42 to 3.61 | 4.94* | 1.40 to 8.48 |
| *Path τ* | |  |  |  |  |  |  |  |  |
|  | Total effects |  |  | 1.76*** | 1.36 to 2.28 | 2.69*** | 2.15 to 3.38 | 5.05*** | 2.61 to 9.79 |
| *Path τ’* | |  |  |  |  |  |  |  |  |
|  | Direct effects |  |  | 1.21 | 0.89 to 1.64 | 1.72*** | 1.30 to 2.28 | 3.54*** | 1.83 to 6.85 |

N for each response: CMD = 7,372; timing of experiences of racism = 8,421; previous infection = 8,276; change in household income = 7,760; financial concerns = 8,152; loneliness and isolation = 8,421; change in loneliness and isolation = 8,303; belonging = 8,138; change in belonging = 8,094.

Reference groups: previous infection = no; change in household income = higher; financial concerns = not at all worried; changes in loneliness = decreased/stopped; belonging = very/fairly strongly; change in belonging = no change.

For indirect effects, statistical significance was determined as confidence intervals without the value of 1.

# **Table S13.** Path analysis estimating the relationship between experiences of interpersonal racism and CMD, through potential pathways (N = 8,391). Analyses are weighted to account for selection bias and coverage bias.

|  | | **CMD** | | **Past experiences** | | **Recent experiences** | | **Past & Recent** | |
| --- | --- | --- | --- | --- | --- | --- | --- | --- | --- |
|  | | **aOR** | **95% CI** | **aOR** | **95% CI** | **aOR** | **95% CI** | **aOR** | **95% CI** |
| *Path α* | |  |  |  |  |  |  |  |  |
|  | Interpersonal 🡪 previous infection *α1* |  |  | 1.00 |  | 1.00 |  | 1.00 |  |
|  | *Yes* |  |  | 1.04 | 0.73 to 1.47 | 1.48** | 1.13 to 1.94 | 0.82 | 0.54 to 1.29 |
|  | Interpersonal 🡪 change in income *α2* |  |  | 1.00 |  | 1.00 |  | 1.00 |  |
|  | *About the same* |  |  | 0.86 | 0.63 to 1.18 | 0.73* | 0.57 to 0.93 | 0.86 | 0.57 to 1.29 |
|  | *Lower* |  |  | 1.11 | 0.79 to 1.56 | 1.06 | 0.81 to 1.39 | 1.41 | 0.94 to 2.13 |
|  | Interpersonal 🡪 financial concerns *α3* |  |  | 1.00 |  | 1.00 |  | 1.00 |  |
|  | *Somewhat concerned* |  |  | 1.23 | 0.92 to 1.66 | 1.52** | 1.20 to 1.92 | 2.01** | 1.35 to 2.99 |
|  | *Very/extremely concerned* |  |  | 1.56* | 1.07 to 2.29 | 2.45*** | 1.80 to 3.34 | 2.97*** | 1.86 to 4.76 |
|  | Interpersonal 🡪 loneliness *α4 (βeta coefficient)* |  |  | 0.63*** | 0.36 to 0.91 | 1.00*** | 0.80 to 1.64 | 1.31*** | 0.99 to 1.64 |
|  | Interpersonal 🡪 change in loneliness *α5* |  |  | 1.00 |  | 1.00 |  | 1.00 |  |
|  | *Stayed the same* |  |  | 0.98 | 0.69 to 1.41 | 0.63** | 0.48 to 0.84 | 1.06 | 0.67 to 1.69 |
|  | *Increased* |  |  | 1.44 | 1.00 to 2.08 | 1.18 | 0.89 to 1.56 | 2.19** | 1.40 to 3.43 |
|  | Interpersonal 🡪 Sense of belonging *α6* |  |  | 1.00 |  | 1.00 |  | 1.00 |  |
|  | *Not very/not at all* |  |  | 1.08 | 0.80 to 1.46 | 1.14 | 0.92 to 1.43 | 1.99*** | 1.40 to 2.85 |
|  | Interpersonal 🡪 change in belonging *α7* |  |  | 1.00 |  | 1.00 |  | 1.00 |  |
|  | *Increased* |  |  | 1.00 | 0.77 to 1.30 | 1.35** | 1.10 to 1.30 | 0.99 | 0.68 to 1.43 |
|  | *Decreased* |  |  | 1.10 | 0.74 to 1.62 | 2.02*** | 1.47 to 2.78 | 1.67* | 1.07 to 2.60 |
| *Path β* | |  |  |  |  |  |  |  |  |
|  | Previous infection 🡪 CMD *β1* | 1.00 |  |  |  |  |  |  |  |
|  | *Yes* | 1.25 | 0.92 to 1.69 |  |  |  |  |  |  |
|  | Change in income 🡪 CMD *β2* | 1.00 |  |  |  |  |  |  |  |
|  | *About the same* | 1.08 | 0.84 to 1.38 |  |  |  |  |  |  |
|  | *Lower* | 1.06 | 0.80 to 1.40 |  |  |  |  |  |  |
|  | Financial concerns 🡪 CMD *β3* | 1.00 |  |  |  |  |  |  |  |
|  | *Somewhat concerned* | 1.84*** | 1.43 to 2.37 |  |  |  |  |  |  |
|  | *Very/extremely concerned* | 4.11*** | 2.91 to 5.82 |  |  |  |  |  |  |
|  | Loneliness 🡪 CMD *β4* | 1.54*** | 1.46 to 1.63 |  |  |  |  |  |  |
|  | Change in loneliness 🡪 CMD *β5* | 1.00 |  |  |  |  |  |  |  |
|  | *Stayed the same* | 0.86 | 0.65 to 1.14 |  |  |  |  |  |  |
|  | *Increased* | 1.63*** | 1.23 to 2.18 |  |  |  |  |  |  |
|  | Sense of belonging 🡪 CMD *β6* | 1.00 |  |  |  |  |  |  |  |
|  | *Not very/not at all* | 1.46** | 1.15 to 1.85 |  |  |  |  |  |  |
|  | Change in belonging 🡪 CMD *β7* | 1.00 |  |  |  |  |  |  |  |
|  | *Increased* | 1.49*** | 1.20 to 1.85 |  |  |  |  |  |  |
|  | *Decreased* | 1.47* | 1.05 to 2.05 |  |  |  |  |  |  |
| *Path α1*β1* | |  |  |  |  |  |  |  |  |
|  | Interpersonal 🡪 CMD (indirect through previous infection) |  |  | 1.00 |  | 1.00 |  | 1.00 |  |
|  | *Yes* |  |  | 1.25 | 0.88 to 1.62 | 1.63* | 1.16 to 2.11 | 1.79 | 1.00 to 2.59 |
| *Path α2*β2* | |  |  |  |  |  |  |  |  |
|  | Interpersonal 🡪 CMD (indirect through change in income) |  |  | 1.00 |  | 1.00 |  | 1.00 |  |
|  | *About the same* |  |  | 1.23 | 0.87 to 1.58 | 1.46* | 1.08 to 1.84 | 1.85* | 1.02 to 2.68 |
|  | *Lower* |  |  | 1.25 | 0.89 to 1.61 | 1.50* | 1.13 to 1.88 | 1.91* | 1.05 to 2.76 |
| *Path α3*β3* | |  |  |  |  |  |  |  |  |
|  | Interpersonal 🡪 CMD (indirect through financial concerns) |  |  | 1.00 |  | 1.00 |  | 1.00 |  |
|  | *Somewhat concerned* |  |  | 1.41 | 0.92 to 1.90 | 1.93* | 1.35 to 2.52 | 2.86* | 1.28 to 4.45 |
|  | *Very/extremely concerned* |  |  | 2.34 | 0.85 to 3.83 | 5.33* | 2.20 to 8.46 | 8.78 | 0.86 to 16.64 |
| *Path α4*β4* | |  |  |  |  |  |  |  |  |
|  | Interpersonal 🡪 CMD (indirect through loneliness) |  |  | 1.64* | 1.12 to 2.15 | 2.32* | 1.71 to 2.92 | 3.31* | 1.81 to 4.81 |
| *Path α5*β5* | |  |  |  |  |  |  |  |  |
|  | Interpersonal 🡪 CMD (indirect through change in loneliness) |  |  | 1.00 |  | 1.00 |  | 1.00 |  |
|  | *Stayed the same* |  |  | 1.25 | 0.89 to 1.61 | 1.60* | 1.14 to 2.07 | 1.85* | 1.05 to 2.67 |
|  | *Increased* |  |  | 1.48 | 0.95 to 2.03 | 1.62* | 1.15 to 2.09 | 2.75* | 1.29 to 4.21 |
| *Path α6*β6* | |  |  |  |  |  |  |  |  |
|  | Interpersonal 🡪 CMD (indirect through belonging) |  |  | 1.00 |  | 1.00 |  | 1.00 |  |
|  | *Not very/not at all* |  |  | 1.28 | 0.89 to 1.67 | 1.58* | 1.16 to 2.00 | 2.43* | 1.21 to 3.64 |
| *Path α7*β7* | |  |  |  |  |  |  |  |  |
|  | Interpersonal 🡪 CMD (indirect through change in belonging) |  |  | 1.00 |  | 1.00 |  | 1.00 |  |
|  | *Increased* |  |  | 1.24 | 0.86 to 1.62 | 1.69* | 1.22 to 2.16 | 4.40* | 1.48 to 7.31 |
|  | *Decreased* |  |  | 1.29 | 0.86 to 1.71 | 1.96* | 1.25 to 2.68 | 2.28* | 1.19 to 3.37 |
| *Path τ* | |  |  |  |  |  |  |  |  |
|  | Total effects |  |  | 1.68*** | 1.31 to 2.14 | 2.31*** | 1.90 to 2.82 | 3.02*** | 2.17 to 4.21 |
| *Path τ’* | |  |  |  |  |  |  |  |  |
|  | Direct effects |  |  | 1.24 | 0.93 to 1.65 | 1.50** | 1.17 to 1.92 | 1.87** | 1.21 to 2.91 |

N for each response: CMD = 7,438; timing of experiences of racism = 8,391; previous infection = 8,364; change in household income = 7,835; financial concerns = 8,237; loneliness and isolation = 8,391; change in loneliness and isolation = 8,391; belonging = 8,220; change in belonging = 8,177.

Reference groups: previous infection = no; change in household income = higher; financial concerns = not at all worried; changes in loneliness = decreased/stopped; belonging = very/fairly strongly; change in belonging = no change.

For indirect effects, statistical significance was determined as confidence intervals without the value of 1.

# **Table S14.** Path analysis estimating the relationship between experiences of institutional racism and CMD, through potential pathways (N = 8,332). Analyses are weighted to account for selection bias and coverage bias.

|  | | **CMD** | | **Past experiences** | | **Recent experiences** | | **Past & Recent** | |
| --- | --- | --- | --- | --- | --- | --- | --- | --- | --- |
|  | | **aOR** | **95% CI** | **aOR** | **95% CI** | **aOR** | **95% CI** | **aOR** | **95% CI** |
| *Path α* | |  |  |  |  |  |  |  |  |
|  | Institutional 🡪 previous infection *α1* |  |  | 1.00 |  | 1.00 |  | 1.00 |  |
|  | *Yes* |  |  | 1.11 | 0.79 to 1.55 | 1.47** | 1.14 to 1.89 | 1.31 | 0.80 to 2.15 |
|  | Institutional 🡪 change in income *α2* |  |  | 1.00 |  | 1.00 |  | 1.00 |  |
|  | *About the same* |  |  | 0.94 | 0.71 to 1.25 | 0.61*** | 0.49 to 0.76 | 0.85 | 0.52 to 1.38 |
|  | *Lower* |  |  | 1.03 | 0.75 to 1.42 | 1.07 | 0.84 to 1.37 | 1.51 | 0.95 to 2.40 |
|  | Institutional 🡪 financial concerns *α3* |  |  | 1.00 |  | 1.00 |  | 1.00 |  |
|  | *Somewhat concerned* |  |  | 1.46** | 1.12 to 1.90 | 1.87*** | 1.50 to 2.34 | 1.29 | 0.82 to 2.03 |
|  | *Very/extremely concerned* |  |  | 2.01*** | 1.43 to 2.82 | 3.29*** | 2.48 to 4.38 | 2.22** | 1.34 to 3.67 |
|  | Institutional 🡪 loneliness *α4 (βeta coefficient)* |  |  | 0.82*** | 0.57 to 1.08 | 1.11*** | 0.91 to 1.30 | 1.46*** | 1.07 to 1.85 |
|  | Institutional 🡪 change in loneliness *α5* |  |  | 1.00 |  | 1.00 |  | 1.00 |  |
|  | *Stayed the same* |  |  | 0.73 | 0.52 to 1.01 | 0.44*** | 0.34 to 0.57 | 0.57* | 0.35 to 0.94 |
|  | *Increased* |  |  | 1.22 | 0.87 to 1.71 | 0.86 | 0.67 to 1.12 | 1.38 | 0.85 to 2.26 |
|  | Institutional 🡪 Sense of belonging *α6* |  |  | 1.00 |  | 1.00 |  | 1.00 |  |
|  | *Not very/not at all* |  |  | 1.01 | 0.77 to 1.32 | 1.22 | 1.00 to 1.49 | 1.70** | 1.15 to 2.50 |
|  | Institutional 🡪 change in belonging *α7* |  |  | 1.00 |  | 1.00 |  | 1.00 |  |
|  | *Increased* |  |  | 1.07 | 0.83 to 1.38 | 1.50*** | 1.24 to 1.82 | 1.45 | 0.95 to 2.22 |
|  | *Decreased* |  |  | 1.17 | 0.85 to 1.63 | 1.99*** | 1.49 to 2.65 | 1.95** | 1.24 to 1.45 |
| *Path β* | |  |  |  |  |  |  |  |  |
|  | Previous infection 🡪 CMD *β1* | 1.00 |  |  |  |  |  |  |  |
|  | *Yes* | 1.23 | 0.90 to 1.67 |  |  |  |  |  |  |
|  | Change in income 🡪 CMD *β2* | 1.00 |  |  |  |  |  |  |  |
|  | *About the same* | 1.09 | 0.85 to 1.41 |  |  |  |  |  |  |
|  | *Lower* | 1.07 | 0.80 to 1.42 |  |  |  |  |  |  |
|  | Financial concerns 🡪 CMD *β3* | 1.00 |  |  |  |  |  |  |  |
|  | *Somewhat concerned* | 1.84*** | 1.43 to 2.38 |  |  |  |  |  |  |
|  | *Very/extremely concerned* | 3.89*** | 2.77 to 5.48 |  |  |  |  |  |  |
|  | Loneliness 🡪 CMD *β4* | 1.54*** | 1.46 to 1.62 |  |  |  |  |  |  |
|  | Change in loneliness 🡪 CMD *β5* | 1.00 |  |  |  |  |  |  |  |
|  | *Stayed the same* | 0.88 | 0.67 to 1.17 |  |  |  |  |  |  |
|  | *Increased* | 1.72*** | 1.29 to 2.30 |  |  |  |  |  |  |
|  | Sense of belonging 🡪 CMD *β6* | 1.00 |  |  |  |  |  |  |  |
|  | *Not very/not at all* | 1.41** | 1.11 to 1.78 |  |  |  |  |  |  |
|  | Change in belonging 🡪 CMD *β7* | 1.00 |  |  |  |  |  |  |  |
|  | *Increased* | 1.46** | 1.18 to 1.82 |  |  |  |  |  |  |
|  | *Decreased* | 1.48* | 1.05 to 2.07 |  |  |  |  |  |  |
| *Path α1*β1* | |  |  |  |  |  |  |  |  |
|  | Institutional 🡪 CMD (indirect through previous infection) |  |  | 1.00 |  | 1.00 |  | 1.00 |  |
|  | *Yes* |  |  | 1.13 | 0.82 to 1.44 | 1.79* | 1.33 to 2.26 | 2.02* | 1.14 to 2.90 |
| *Path α2*β2* | |  |  |  |  |  |  |  |  |
|  | Institutional 🡪 CMD (indirect through change in income) |  |  | 1.00 |  | 1.00 |  | 1.00 |  |
|  | *About the same* |  |  | 1.10 | 0.80 to 1.40 | 1.59* | 1.17 to 2.01 | 1.88* | 1.04 to 2.73 |
|  | *Lower* |  |  | 1.11 | 0.81 to 1.41 | 1.67* | 1.28 to 2.06 | 1.97* | 1.07 to 2.86 |
| *Path α3*β3* | |  |  |  |  |  |  |  |  |
|  | Institutional 🡪 CMD (indirect through financial concerns) |  |  | 1.00 |  | 1.00 |  | 1.00 |  |
|  | *Somewhat concerned* |  |  | 1.39 | 0.93 to 1.86 | 2.44* | 1.66 to 3.21 | 2.23 | 0.97 to 3.49 |
|  | *Very/extremely concerned* |  |  | 2.86* | 1.14 to 4.57 | 8.39* | 3.38 to 13.41 | 5.64 | 0.63 to 10.65 |
| *Path α4*β4* | |  |  |  |  |  |  |  |  |
|  | Institutional 🡪 CMD (indirect through loneliness) |  |  | 1.58* | 1.12 to 2.05 | 2.68* | 2.01 to 3.35 | 3.59* | 1.87 to 5.32 |
| *Path α5*β5* | |  |  |  |  |  |  |  |  |
|  | Institutional 🡪 CMD (indirect through change in loneliness) |  |  | 1.00 |  | 1.00 |  | 1.00 |  |
|  | *Stayed the same* |  |  | 1.15 | 0.82 to 1.48 | 1.84* | 1.22 to 2.45 | 2.05* | 1.03 to 3.06 |
|  | *Increased* |  |  | 1.24 | 0.82 to 1.65 | 1.53* | 1.08 to 1.98 | 2.28* | 1.19 to 3.37 |
| *Path α6*β6* | |  |  |  |  |  |  |  |  |
|  | Institutional 🡪 CMD (indirect through belonging) |  |  | 1.00 |  | 1.00 |  | 1.00 |  |
|  | *Not very/not at all* |  |  | 1.11 | 0.79 to 1.43 | 1.78* | 1.34 to 2.21 | 2.29* | 1.18 to 3.40 |
| *Path α7*β7* | |  |  |  |  |  |  |  |  |
|  | Institutional 🡪 CMD (indirect through change in belonging) |  |  | 1.00 |  | 1.00 |  | 1.00 |  |
|  | *Increased* |  |  | 1.14 | 0.81 to 1.47 | 1.94* | 1.44 to 2.43 | 2.20* | 1.11 to 3.29 |
|  | *Decreased* |  |  | 1.18 | 0.82 to 1.54 | 2.17* | 1.46 to 2.88 | 2.48* | 1.24 to 3.72 |
| *Path τ* | |  |  |  |  |  |  |  |  |
|  | Total effects |  |  | 1.70*** | 1.36 to 2.12 | 2.62*** | 2.17 to 3.15 | 3.16*** | 2.19 to 4.57 |
| *Path τ’* | |  |  |  |  |  |  |  |  |
|  | Direct effects |  |  | 1.11 | 0.85 to 1.45 | 1.66*** | 1.32 to 2.09 | 1.91** | 1.23 to 2.98 |

N for each response: CMD = 7,397; timing of experiences of racism = 8,332; previous infection = 8,306; change in household income = 7,786; financial concerns = 8,179; loneliness and isolation = 8,332; change in loneliness and isolation = 8,332; belonging = 8,167; change in belonging = 8,124.

Reference groups: previous infection = no; change in household income = higher; financial concerns = not at all worried; changes in loneliness = decreased/stopped; belonging = very/fairly strongly; change in belonging = no change.

For indirect effects, statistical significance was determined as confidence intervals without the value of 1.
